# Delineating the mechanism of anti-Lassa virus GPC-A neutralizing antibodies

**DOI:** 10.1016/j.celrep.2022.110841

**Published:** 2022-05-24

**Authors:** Adrian S. Enriquez, Tierra K. Buck, Haoyang Li, Michael J. Norris, Alex Moon-Walker, Michelle A. Zandonatti, Stephanie S. Harkins, James E. Robinson, Luis M. Branco, Robert F. Garry, Erica Ollmann Saphire, Kathryn M. Hastie

**Affiliations:** 1 La Jolla Institute for Immunology, La Jolla, CA 92037, USA; 2 Program in Virology, Harvard University, Boston, MA 02115, USA; 3 Department of Molecular Microbiology, Washington University in Saint Louis, St. Louis, MO 63130, USA; 4 Department of Pediatrics, Tulane University School of Medicine, New Orleans, LA 70112, USA; 5 Zalgen Labs, LLC, Germantown, MD 20876, USA; 6 Department of Microbiology and Immunology, Tulane University School of Medicine, New Orleans, LA 70112, USA; 7 Lead contact

## Abstract

Lassa virus (LASV) is the etiologic agent of Lassa Fever, a hemorrhagic disease that is endemic to West Africa. During LASV infection, LASV glycoprotein (GP) engages with multiple host receptors for cell entry. Neutralizing antibodies against GP are rare and principally target quaternary epitopes displayed only on the metastable, pre-fusion conformation of GP. Currently, the structural features of the neutralizing GPC-A antibody competition group are understudied. Structures of two GPC-A antibodies presented here demonstrate that they bind the side of the pre-fusion GP trimer, bridging the GP1 and GP2 subunits. Complementary biochemical analyses indicate that antibody 25.10C, which is broadly specific, neutralizes by inhibiting binding of the endosomal receptor LAMP1 and also by blocking membrane fusion. The other GPC-A antibody, 36.1F, which is lineage-specific, prevents LAMP1 association only. These data illuminate a site of vulnerability on LASV GP and will guide efforts to elicit broadly reactive therapeutics and vaccines.

## In brief

Enriquez et al. present two structures of GPC-A antibody Fab fragments bound to Lassa virus glycoprotein. Complementary biochemical analyses illuminate mechanistic differences between pan-Lassa 25.10C and lineage-specific 36.1F. 25.10C inhibits two steps of Lassa virus infection, LAMP1 binding and membrane fusion, while 36.1F only blocks LAMP1.

## Graphical Abstract

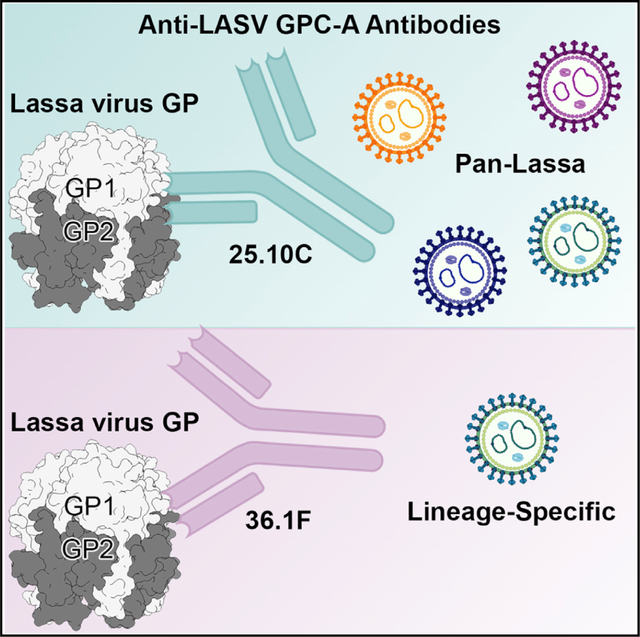

## INTRODUCTION

The causative agent of Lassa Fever (LF) is Lassa virus (LASV, family Arenaviridae), a highly lethal enveloped virus that infects hundreds of thousands of people across multiple countries in West Africa (McCormick and Fisher-Hoch, 2002) and causes widespread social and economic disruption (Shaffer et al., 2014). LASV is transmitted to humans primarily through contact with excretions from rodent reservoirs of LASV, including the common peridomestic rodent *Mastomys natalensis* and other newly identified rodent hosts (Monath et al., 1974; Demby et al., 2001; Fichet-Calvet et al., 2008; Olayemi et al., 2016). One challenge for developing a vaccine against LASV is its broad genetic diversity (Andersen et al., 2015; Heinrich et al., 2020). There are seven distinct LASV lineages (LI–LVII) that are classified according to their geographic origin and phylogeny (Bowen et al., 2000; Andersen et al., 2015; Manning et al., 2015; Oloniniyi et al., 2018; Whitmer et al., 2018; Ehichioya et al., 2019; Heinrich et al., 2020). Currently, there is no Food and Drug Administration-approved therapeutic against LASV. The only available treatment for LF is the antiviral ribavirin, which is only effective during early infection (McCormick et al., 1986). Around 30% of patients who survive LF develop lifelong uni- or bilateral sensorineural hearing sequelae that manifest during convalescence (Cummins et al., 1990; Cashman et al., 2018).

The LASV glycoprotein (GP), the sole protein exposed on the virion surface, is the primary target of the humoral antibody response (Sommerstein et al., 2015). The mature, trimeric GP protrudes from the viral membrane as a tripartite complex comprising the stable signal peptide (SSP), the receptor binding subunit GP1, and the transmembrane fusion subunit GP2 (Eichler et al., 2003). Mature GP is heavily glycosylated, with 11 highly conserved N-linked glycosylation sites, seven in GP1 and four in GP2, that accommodate dense patches of oligomannose-rich carbohydrates, which account for 25% of the total GP mass (Watanabe et al., 2018). The extensive glycosylation of GP leaves few regions exposed and is a critical component for viral evasion of the humoral response (Sommerstein et al., 2015).

LASV infection is initiated by engagement of GP1 with matriglycosylated α-dystroglycan (α-DG) (Cao et al., 1998; Kunz et al., 2005; Inamori et al., 2012; Yoshida-Moriguchi and Campbell, 2015; Acciani et al., 2017). This interaction facilitates uptake of LASV by macropinocytosis (Cao et al., 1998; Jae et al., 2014; Oppliger et al., 2016), and occurs most efficiently at alkaline pH. Upon reaching the mildly acidic interior of early endosomal compartments, GP undergoes a pH-dependent intracellular receptor switch to disengage from α-DG and bind to lysosome-associated membrane protein 1 (LAMP1). This receptor shift promotes fusion between host and virus membranes (Jae et al., 2014). Binding of LAMP1 with GP involves histidines H92, H93, and H230 on GP1 that act as pH sensors (Cohen-Dvashi et al., 2015). In particular, protonation of H230 at endosomal pH ≤ 6.0 promotes the LAMP1-GP interaction, which drives fusion of host and virus membranes (Cohen-Dvashi et al., 2016). Fusion of LASV and target cell membrane can occur without LAMP1, but is inefficient and occurs in very late endosomes (Hulseberg et al., 2018).

Neutralizing antibodies against LASV GP are rare and for decades had evaded detection. With few exceptions, binding of LASV neutralizing antibodies requires the presentation of quaternary epitopes that are only displayed on the metastable, pre-fusion conformation of GP (Robinson et al., 2016). We recently characterized the putative epitopes of a panel of 113 monoclonal antibodies (mAbs) from the memory B cells of West African patients who had survived LF and found just 16 mAbs that neutralize LIV (Josiah strain) LASV (Robinson et al., 2016). These neutralizing antibodies were categorized into four competition groups, termed GPC-A, GPC-B, GPC-C, and GP1-A, with the majority comprising the GPC-B competition group. To date, only antibodies in the GPC-B competition group have been structurally analyzed and their respective epitopes mapped to high resolution (Hastie et al., 2017, 2019). Further, many GPC-B antibodies are ineffective against lineage I of LASV (Hastie et al., 2019). A comprehensive understanding of all sites of vulnerability on LASV GP and how antibodies evolve to recognize these sites is valuable for future immunogen design. Moreover, determination of the exact molecular interface on GP targeted by antibodies in different competition groups will provide additional insight into the mechanisms of immune surveillance and evasion by LASV.

In this study, we present high-resolution epitope maps of the GPC-A mAbs 25.10C and 36.1F determined using cryoelectron microscopy (cryo-EM) and X-ray crystallography, respectively; 25.10C is a highly potent and pan-LASV mAb, while 36.1F is moderately potent and lineage IV-specific (Robinson et al., 2016). The two structures presented here highlight the molecular differences between these two GPC-A mAbs that target overlapping regions on GP but exhibit contrasting LASV lineage specificity. Furthermore, using complementary biochemical approaches, we identify likely mechanisms involved in virus neutralization by 25.10C and 36.1F. Delineating the molecular details governing GPC-A mAb-mediated neutralization sheds light on the targetable surfaces on LASV GP that are critical for developing a cost-effective, potent, and widely available treatment for LASV infection.

## RESULTS

### Structure determination of pre-fusion LASV GP in complex with GPC-A antibodies

The *in vitro* instability of the pre-fusion LASV GP trimer was an obstacle to high-resolution structural analyses. Recently, our team successfully engineered an LASV GP ectodomain, GPCysR4, which is kinetically arrested in the pre-fusion conformation and is also recognized by neutralizing antibodies (Hastie et al., 2017). GPCysR4 (referred to here as pfGP) is purified as a furin-processed monomeric species of GP1 and GP2 subunits (without SSP) linked by a disulfide bond. Upon incubation with GPC-B Fab fragments, the pfGP ectodomain exists in an equilibrium of monomeric and trimeric Fab-pfGP complexes. In contrast to GPC-B mAbs, GPC-A mAbs do not bridge adjacent monomers in the trimer, but rather span GP1 and GP2 subunits within a single monomer; GPC-A mAbs on their own do not induce pfGP trimerization. To overcome this limitation, the GPC-B 18.5C Fab, which binds lower on the pfGP trimer relative to GPC-A antibodies, was used as a crystallization chaperone for 36.1F-pfGP complexes. The 18.5C-36.1F-pfGP ternary complexes formed crystals in the space group P321 that diffracted to 2.7 Angstroms (Å) (Table S1). Application of crystallographic symmetry revealed that the pfGP trimer bound three 18.5C Fabs and three 36.1F Fabs (Figures S1A and S1B).

We also attempted to form 18.5C-25.10C-pfGP ternary complexes, but could not obtain monodisperse ternary complexes with LASV pfGP simultaneously bound to both Fabs. Attempts to crystallize 25.10C in complex with pfGP monomers were similarly unsuccessful. To overcome these hurdles, we enzymatically ligated monomeric pfGP to an exogenous trimerization domain (PDB: 1NOG, protein termed pfGP-TD) (Saridakis et al., 2004) prior to incubation with 25.10C Fab. High-resolution structural data were obtained from 25.10C-pfGP-TD complexes under cryo-EM conditions that resulted in a 3.0Å 3-D reconstruction with three 25.10C Fabs bound to pfGP-TD (Figures S1C–S1E and S2, and Table S2). Local resolution estimates of the cryo-EM reconstruction indicate that the 25.10C-pfGP-TD complex is conformationally labile. The strongest and higher-resolution density corresponds to the Fab-pfGP interface, while the lower resolution density corresponds to the Fab CH/CL domain (Figure S3A).

### Constricting the elbow region allows crystallization of the 25.10C Fab

Accurate modeling of the epitopes recognized by GPC-A antibodies is critical for future vaccine and improved therapeutic designs. Although the Fab-GP interface in the 25.10C-pfGP-TD cryo-EM reconstruction contains interpretable amino acid side chain density, we also deciphered the 25.10C Fab paratope using X-ray crystallography. Initially, wild-type 25.10C Fab obtained through papain digestion of immunoglobulin (Ig)G did not yield crystals. The weaker density of the Fab CH/CL domains observed in our 25.10C-GP cryo-EM reconstruction suggests that the presence of a potential flexible elbow joint between the 25.10C constant and variable domains could impede packing of the Fab into a crystal lattice. To restrict the number of possible elbow angles, we introduced a single residue deletion coupled with a bulky, four-amino acid substitution to the heavy chain elbow domain (ΔS_112_ and S_113_AST_116_ to F_113_NQI_116_) (Bailey et al., 2018). Crystals of 25.10C-FNQI grew in space group P12_1_1 and diffracted to 2.6 Å (Table S3). The complementarity determining regions (CDRs) for both the heavy and light chain of 25.10C had clear density and could be completely modeled. Interestingly, a domain swap occurred between the CH domains of two 25.10C Fabs, resulting in a dimer that comprises the asymmetric unit (Figure S3B). A similar phenomenon was previously reported for the anti-HIV antibody 2G12, which involves a domain-swapped dimeric conformation as part of its mechanistic action in solution (Calarese et al., 2003; Stanfield et al., 2015). In contrast, the 25.10C dimer observed here is likely due to the elbow mutation and crystal packing, as no evidence for a Fab dimer is observed in the cryo-EM data with wild-type 25.10C Fab.

### Divergent germlines converge on the same quaternary epitope

**Figure 1.**
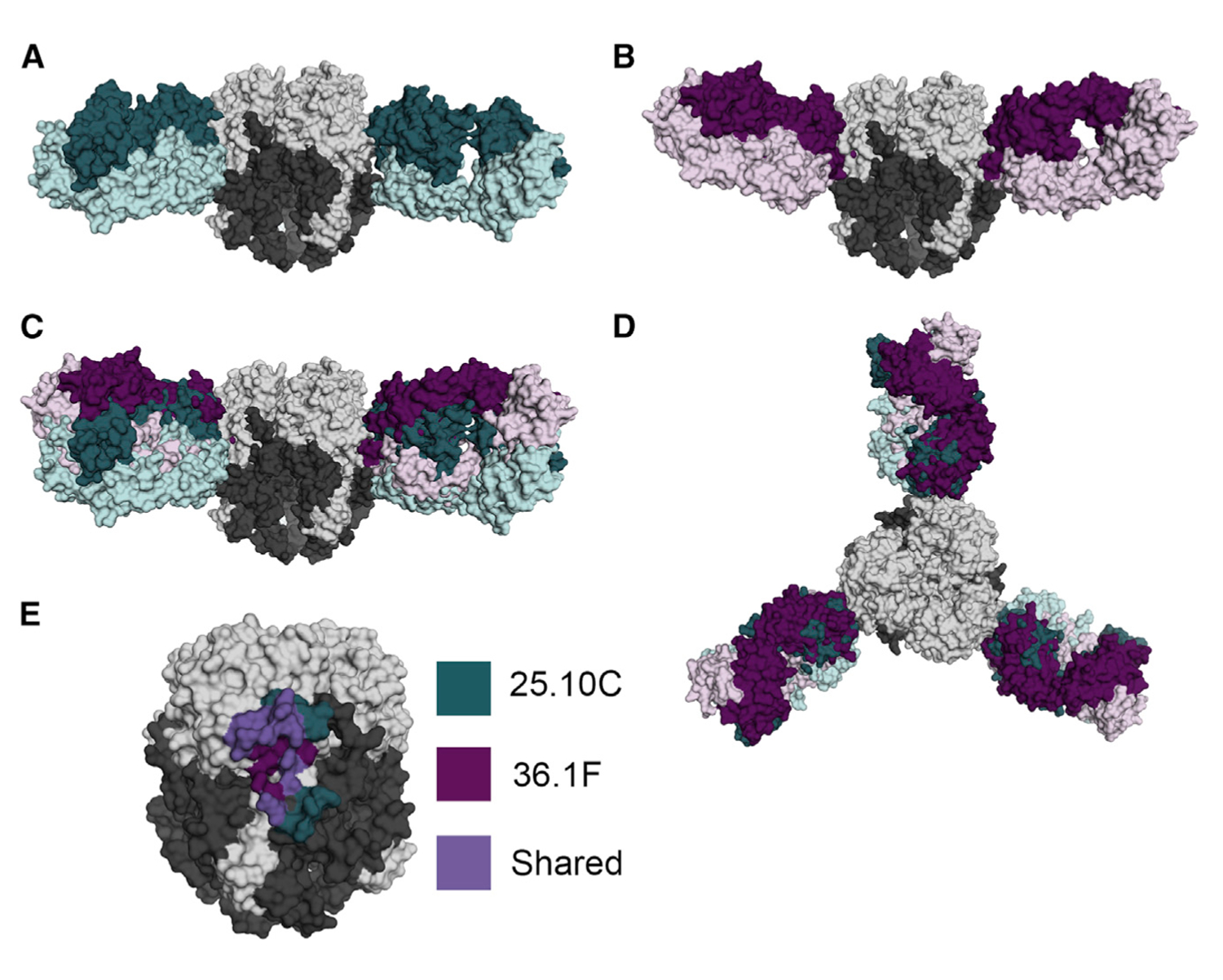
Structural elucidation of the GPC-A epitope (A) Side view of the 25.10C-pfGP atomic model as a surface representation. GP1 and GP2 are colored light and dark gray, respectively. The light and heavy chain domains of the 25.10C Fab are colored light and dark green, respectively. (B) Side view of the 18.5C-36.1F-pfGP ternary complex (only 36.1F is shown for clarity). The light and heavy chains of the 36.1F Fab are colored light and dark magenta, respectively. (C) Side view of superposed 25.10C- and 36.1F-pfGP complexes depicting the overlapping quaternary GPC-A epitope. (D) Top view along the trimeric axis showing the overlapping GPC-A epitope. (E) The antigenic landscape of the GPC-A antibody competition group. Residues in pfGP contacted by 25.10C are shown in dark green and those contacted by 36.1F are in dark magenta. Residues contacted by both Fabs are in purple. See also Figures S1, S2, S3A, and S3B, and Tables S1, S2, and S3.

**Figure 2.**
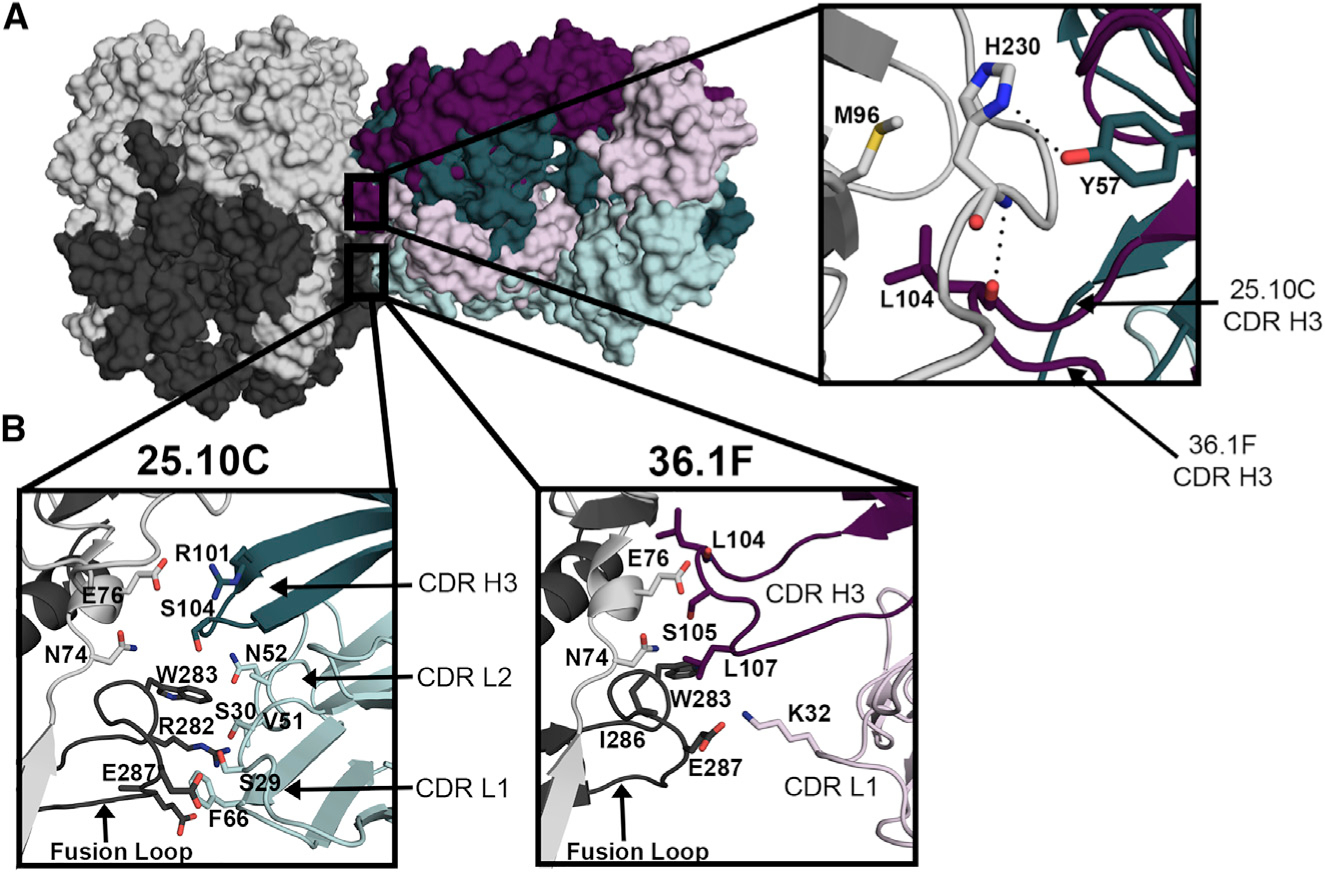
GPC-A antibodies share an overlapping, yet distinct epitope (A) Overlay of the 25.10C and 36.1F heavy chain CDRs depicting different contacts with GP1 residue H230: 25.10C contacts H230 head-on via Y57 of the CDR-H2, whereas for 36.1F L104 in the longer CDR-H3 contacts from behind H230. (B) The heavy chain CDR-H3 and light chain CDRs-L1 and -L2 of 25.10C make extensive contacts to the fusion loop of GP2 (residues 270–292). In contrast, 36.1F makes just two contacts with the fusion loop: a hydrophobic interface between W283 of GP2 and L107 of CDR-H3 and a hydrogen bond between E287 and CDR-L1 residue K32. See also Figures S3C, S3D, S4, and S5A.

Unlike GPC-B antibodies that typically share the VH3–21 and VK3–20 germlines, regardless of whether they were isolated from the same or different survivors, the GPC-A antibodies 25.10C and 36.1F are derived from different heavy and light chain germlines; 25.10C is derived from VH3–21 and VK1–39 heavy and light chains, whereas 36.1F derives from VH4–34 and VK3–20 heavy and light chains (Figures S3C–S3D). Both antibodies approach the pfGP protomer from the side and bridge GP1 and GP2 monomers through a quaternary epitope (Figures 1A–1D). Both antibodies also bury a similar amount of surface area on pfGP (~650–680 Å). However, the 25.10C epitope is distributed over the GP1 (440Å) and GP2 (240 Å) subunits, while the 36.1F epitope is dominated by GP1 contacts, with only a small (40 Å) region contacting GP2. The majority of the shared residues between the two GPC-A mAbs are located in GP1 (Figure 1E), and includes N74, E76, K88, T226, W227, E228, D229, H230, and Q232. Of these, residues 226 to 232 in GP1 are located on the β-sheet face near the GP1-GP2 interface and are part of a flexible loop that forms an array of hydrogen bonds with both antibodies. This loop presumably undergoes a conformational change during the transition from the pre-fusion to the primed state of GP1 that occurs upon protonation of the GP1 residue H230 (Cohen-Dvashi et al., 2015; Hastie et al., 2017). Although both antibodies bind pfGP in a similar location, 25.10C and 36.1F contact this loop and H230 in particular via different CDRs and angles of approach. The hydroxyl of Y57 in the 25.10C CDR-H2 forms a hydrogen bond to the ND1 nitrogen of the H230 imidazole side chain in a head-on manner. The longer CDR-H3 of 36.1F instead reaches behind the loop to form a hydrogen bond between the main-chain oxygen of L104 with the main-chain nitrogen of H230 (Figure 2A).

While multiple contact points in GP1 are shared by 25.10C and 36.1F, there are no shared contacts between the two antibodies in GP2. For 25.10C, CDR-L2 G65 forms a hydrogen bond with R282 in GP2, and hydrophobic interactions with the CDR-L1 and FR-2 domains completely sequester GP2 residues 282 to 289. Meanwhile, for 36.1F, CDR-L1 residue K32 forms a salt bridge with GP2 residue E287, and the adjacent GP2 residue I286 makes a hydrophobic interaction with CDR-H3 residue L107 (Figures 2B and S4).

The pfGP fusion peptide (residues 261–279) is conformationally labile and adopts various conformations when bound to different GPC-B mAbs (Hastie et al., 2017, 2019). Due to the absence of contacts between 25.10C and the fusion peptide, there was an unresolvable density for this region in cryo-EM analyses. Hence, only the fusion loop (residues 280–292), which is stabilized by several Fab contacts, could be modeled. In the 36.1F structure, both the fusion loop and fusion peptide are well delineated, ostensibly from co-crystallizing as a ternary complex with 18.5C, which targets an epitope that encompasses the fusion loop (Hastie et al., 2019).

### GP1 and GP2 subunit glycans in the GPC-A epitope

Both Fab-GP structures reveal that the GPC-A epitope is nestled between GP1 glycans N79, N99, and N224 that surround the heavy chains, and GP1 glycan N89 and GP2 glycan N365 that surround the light chains (Figure S5). These glycans, except that at N224, were previously shown to comprise a spatially dense oligomannose cluster that sterically obstructs access to the LAMP1 binding site in pre-fusion GP1 and in the fusion loop of GP2 (Watanabe et al., 2018). Specifically, glycan N89 conceals H92 and H93 of the LAMP1 histidine triad in GP1, but leaves H230 unsequestered and accessible (Watanabe et al., 2018). Both steric and functional constraints on the pre-fusion H230 loop likely hinder the accumulation of glycans at this region. This possibility is supported by the high conservation and lack of deletions or additions of glycosylation sites in GP1 across LASV lineages. Notably, glycan N79, N99, and N365 are required for proper processing of LASV GPC (Eichler et al., 2006) and *in vitro* virion rescue (Bonhomme et al., 2013). The finding that both 25.10C and 36.1F converged on one of the few accessible regions within the oligomannose cluster indicates that the GPC-A epitope is both highly immunogenic and subject to immune pressure.

### The CDR-H3 of 36.1F contacts multiple non-conserved residues in GP1

**Figure 3.**
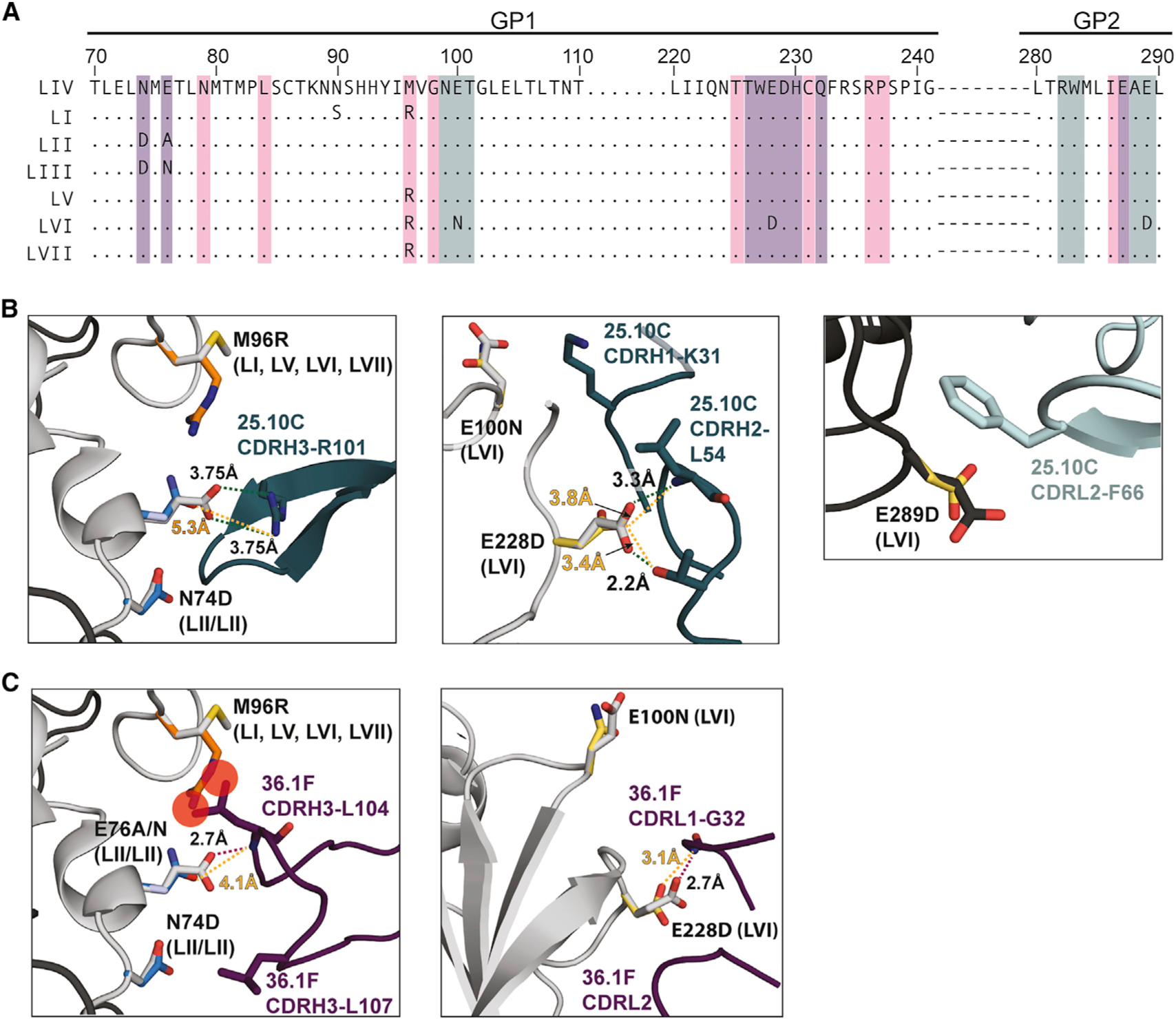
Structural impact of natural amino acid substitutions in the GPC-A epitope found in various LASV lineages (A) Sequence alignment of LASV lineages. Dots indicate consensus residues. Residues in LIV GP contacted by 25.10C are shaded cyan and those contacted by 36.1F are shaded magenta. Residues contacted by both 25.10C and 36.1F are shaded purple. (B) (Left) Key interactions between CDRH3 of 25.10C (cyan) and LASV GP. The identity of the residue in LASV LIV is shown in gray with the variant residue colored orange (LI, LV, LVI, and LVII variations) or blue (LII and LIII variations). Substitution of M96 with arginine would not affect the short CDR-H3 of 25.10C. Residue E76 in LASV LIV forms a salt bridge with 25.10C R101 (green dotted lines). Interaction between A76 and 25.10C R101 would not occur in lineage LII. Interaction with N76 (dark blue) of LIII and this residue may be possible with a shift in the R101 rotamer (light cyan). LII and LIII also have an N74D variation, but this is unlikely to substantially impact binding. (Middle) Interactions between 25.10C CDR-H1 and CDR-H2 with LASV GP. The identity of the residue in LASV LIV is shown in gray with the LVI variant residue colored yellow. Substitution of LIV E100 with asparagine would likely result in a similar interaction between 25.10C CDR-H1 K31. Similarly, substitution of E228 with aspartic acid would permit a similar interaction. (Right) The substitution of E289 to aspartic acid (yellow) would likely maintain the aliphatic interaction with 25.10C CDR-L2 F66. (C) (Left) Key interactions between 36.1F (magenta) and LASV GP. Substitution of M96 with arginine likely results in a steric clash with 36.1F CDRH3 residue L104 (overlapping red circles). Residue E76 in LASV LIV forms a hydrogen bond with the main-chain nitrogen of 36.1F CDR-H3 L104 (purple dotted lines) and substitution with alanine (LII) or asparagine (LIII), which have shorter side chains, would no longer hydrogen bond 36.1F L104. (Middle) The LASV LVI E100 N substitution (yellow) would have no effect on binding by 36.1F since no contact is made to residue E100 in LIV. Similarly, the E228D substitution would have little effect on 36.1F since D228 (yellow) is still capable of hydrogen bonding 36.1F CDR-H1 G32. For (B) and (C), amino acid substitutions were generated in PyMOL using the mutagenesis wizard. Backbone-dependent rotamers were selected that have minimal steric clashes and best mimic the rotamer of the amino acid being substituted.

To better understand why 36.1F is lineage specific and why 25.10C maintains pan-LASV specificity, we modeled the natural amino acid substitutions present in various LASV lineages onto the LIV-pfGP structure (Figure 3A). Differences in breadth of recognition are likely due to differences in the particular residues contacted by the CDR H3 of each antibody in the overlapping footprints. The CDRH3 of 36.1F contacts GP1 residues N74, E76, and M96, which are poorly conserved across LASV lineages (Figure 3C, left). Of these three residues, the 25.10C CDR H3 only contacts one: E76 via a salt bridge with its CDR H3 R101 (Figure 3B, left). Mutation of E76 to alanine (LII) would prevent such a salt bridge, but asparagine (LIII) may still form a hydrogen bond. For 361.F, the hydrogen bond between GP E76 and the main-chain of L104 of 36.1F CDRH3 would also likely not form in the E76N variation of LIII and would be impossible in E76A of LII. Furthermore, modeling of the M96R substitution present in lineages LI, LV, LVI, and LVII suggests that the larger arginine side chain would sterically clash with CDR-H2 residue L104 in 36.1F (Figure 3C, left). LASV LVI also has E100 N, E228D, and E289D variations that are not present in other lineages. These substitutions are unlikely to have an impact on binding to either antibody (Figures 3B and 3C).

Taken together, these data indicate that 36.1F-mediated LASV neutralization, which involves multiple contacts at non-conserved GP1 residues, is more susceptible to inter-lineage variations in GP than is 25.10C. Indeed, little difference in neutralization potency by 25.10C is observed across neutralization of LI-LIV LASV pseudovirions (Robinson et al., 2016).

### GPC-A mAbs block interaction with LAMP1

**Figure 4.**
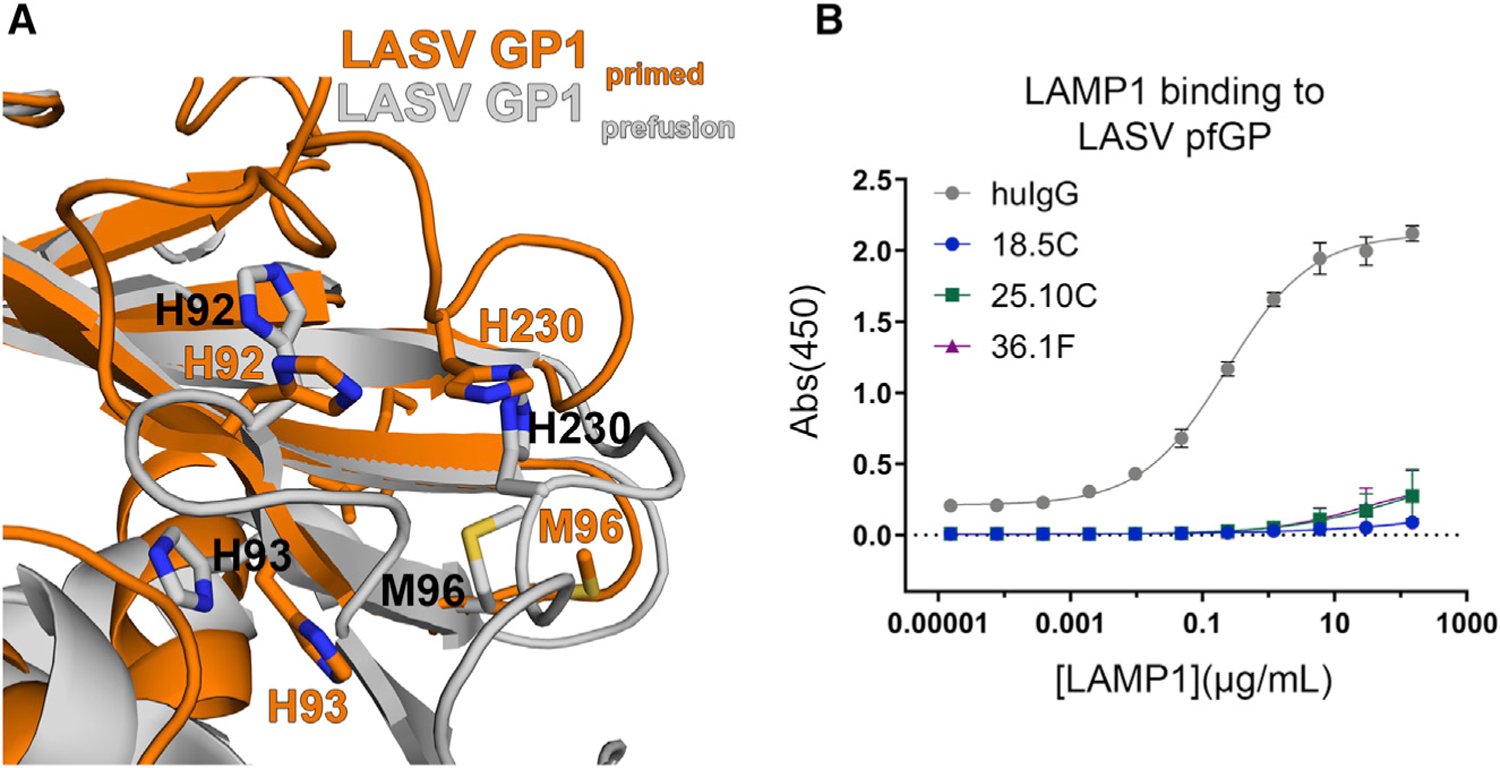
GPC-A antibodies prevent association with LAMP1 (A) Superimposition of the GP1 subunit of LASV GP in the pre-fusion (gray cartoon) and primed (orange) conformation highlights structural rearrangements that may occur upon LAMP1 binding. The loop harboring H230 is central to the GPC-A epitope and its location and its location shifts ~90° relative to the pre-fusion state. (B) ELISA analysis of LAMP1 association with LASV pfGP-TD alone and in complex with the indicated IgG. Error bars indicate the mean ± SD of two independent experiments, each performed in duplicate.

The binding site of the LAMP1 receptor has not yet been characterized structurally, but is thought to involve a loop in GP1 comprising residues 225 to 232 and including pH-sensing residue H230 (Cohen-Dvashi et al., 2016). LAMP1 binding occurs in the low pH of the endosome. Superimposition of the crystal structure of GP1 solved at pH 5.0, with the GP1 of the pfGP trimer solved at neutral pH reveals differences in the location of H230 (Cohen-Dvashi et al., 2015; Hastie et al., 2017). In response to the low pH of the endosome, GP1 presumably undergoes conformational changes that occur prior to or concomitant with LAMP1 binding. Hence, one mechanism for neutralization by these antibodies may be to block access to the H230-containing loop or to lock it in a pre-fusion conformation to prevent its association with LAMP1. The structures of 25.10C and 36.1F in complex with GP indicate that both antibodies bind to this loop, and when bound, would physically block LAMP1 binding. In ELISA, pre-incubation of either 25.10C or 36.1F with GP indeed prevents subsequent binding of GP to LAMP1 (Figure 4B). We note, however, the epitope of 25.10C is shifted lower than that of 36.1F such that 25.10C also recognizes the fusion loop in GP2, while 36.1F does not. Hence, it was possible that 25.10C could exhibit a different mechanism of neutralization.

### LAMP1 and pH independence (25.10C) and dependence (36.1F)

**Figure 5.**
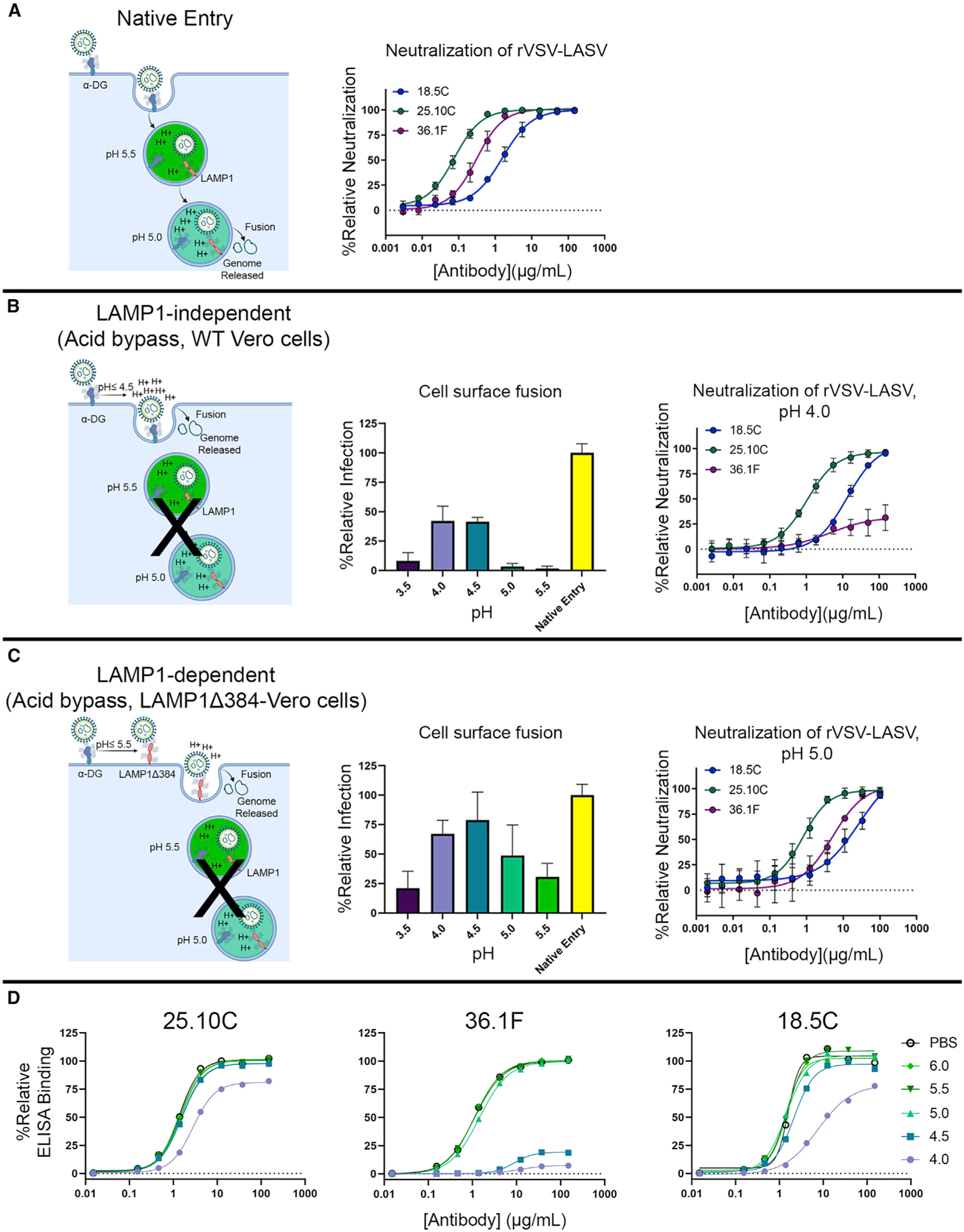
LAMP-1 independent manner neutralization of rVSV-LASV by the GPC-A mAb 25.10C (A) Schematic depicting native entry and fusion of LASV into cells. GP interacts with α-DG at the cell surface and undergoes a pH-dependent receptor shift to LAMP-1 inside the endosome. The pH 5.0 environment of the late endosome promotes membrane fusion and subsequent release of the viral genome. (Middle) Neutralization of rVSV-LASV by the GPC-A mAbs 25.10C and 36.1F and the GPC-B mAb 18.5C in wild-type Vero cells under native entry conditions. (B) (Left) Schematic depicting entry of LASV at the cell surface in wild-type Vero cells. In this entry pathway, exposure to acidic pH, or acid bypass, forces fusion of target cell and LASV membranes; this fusion occurs in a LAMP1-independent manner. (Middle) Fusogenic profile of rVSV-LASV at the indicated pH. (Right) Neutralization of rVSV-LASV by the GPC-A mAbs 25.10C and 36.1F and the GPC-B mAb 18.5C in wild-type Vero cells, at the cell surface at pH 4.0. (C) (Left) Schematic depicting entry of LASV at the cell surface in Vero cells expressing LAMP1Δ384 with cell-surface localization of LAMP1. In this entry pathway, LASV first binds to cell-surface receptors, such as matriglycosylated α-DG and then, upon exposure to lower pH, switches to cell-surface LAMP1Δ384 before fusion. (Middle) The fusogenic profile of rVSV-LASV in LAMP1Δ384-Vero cells at the indicated pH is shown. (Right) Neutralization of rVSV-LASV by the GPC-A mAbs 25.10C and 36.1F and the GPC-B mAb 18.5C in Vero cells stably expressing LAMP1Δ384 at pH 5.0. (D) (Left, middle, right) Binding of the indicated antibody to LASV pfGP-TD at varying pHs as determined by ELISA. For (B-middle) and (C-middle), data were normalized to the total infectivity of virus under native entry conditions (i.e., endosomal dependent, and not exposed to either acid or lysosomotropic agents). Error bars indicate the mean ± SD of two independent experiments, each performed in quadruplicate. For (A-middle), (B-right), and (C-right), data are displayed as the relative neutralization as compared with a PBS control. Error bars indicate the SD of the mean of three independent experiments, each performed in duplicate. Schematics were created with BioRender.com. See also Figure S6.

To understand if blocking the GP-LAMP1 interaction is the primary mechanism of neutralization for GPC-A antibodies, we investigated the ability of 25.10C and 36.1F to neutralize infection by rVSV-LASV-GP in LAMP1-dependent and -independent manners. We first made a Vero cell line that stably expresses LAMP1Δ384 (Figure S6). The Δ384 mutation results in ectopic localization of LAMP1 to the cell surface, instead of only in the endosome, so that we could measure neutralization in the presence or absence of LAMP1 and at different pHs (Rohrer et al., 1996). We then compared the fusogenic profile at various pHs of rVSV-LASV-GP with LAMP1Δ384-transduced (LAMP1-dependent) and not transduced (LAMP1-independent) cells. In the absence of LAMP1 at the cell surface, as would be typical, and consistent with previous studies, fusion required pH ≤ 4.5 (Figure 5B, middle). In the presence of LAMP1, achieved by transduction with LAMP1Δ384, fusion occurred at a higher pH threshold, with robust fusion at pH ≤ 5.5 (Figure 5C, middle). This result confirms that cell-surface LAMP1 increases the possible pH at which fusion between virus and target cell membranes can occur, and offers a system in which we could evaluate antibody neutralization in the presence or absence of LAMP1 and at different pH.

At pH 5.0, where fusion is LAMP1-dependent, and in the presence of cell-surface LAMP1, both 25.10C and 36.1F neutralize infection (Figure 5B, right). At pH 4.0, where fusion is LAMP1-independent, and in the absence of cell-surface LAMP1, 25.10C could neutralize virus infection, while 36.1F could not (Figure 5C, right). Hence, 25.10C can neutralize at pH 4.0 and in a LAMP1-independent manner.

The loss of neutralization activity by 36.1F at pH 4.0 could be due to pH-dependent loss of 36.1F binding to GP, whether or not LAMP1 was present. To examine this possibility, we assessed the binding of GPC-A mAbs to trimeric pfGP under neutral and increasingly acidic conditirons. We found that binding of 25.10C to pfGP is relatively pH insensitive: at pH 4.0, 25.10C maintains ~75% of binding to pfGP compared with that observed at pH ≤ 4.5. This profile is similar to that for the GPC-B mAb 18.5C, which binds mostly GP2 (Figure 5D, left, right). In contrast, 36.1F association with pfGP is markedly more pH sensitive, with near total loss of binding at pH < 5.0 (Figure 5D, middle). Hence, while 25.10C can likely bind to GP even in late endosomal compartments, 36.1F may bind GP only at the cell surface and in compartments in which the pH is higher than 5.0.

**Figure 6.**
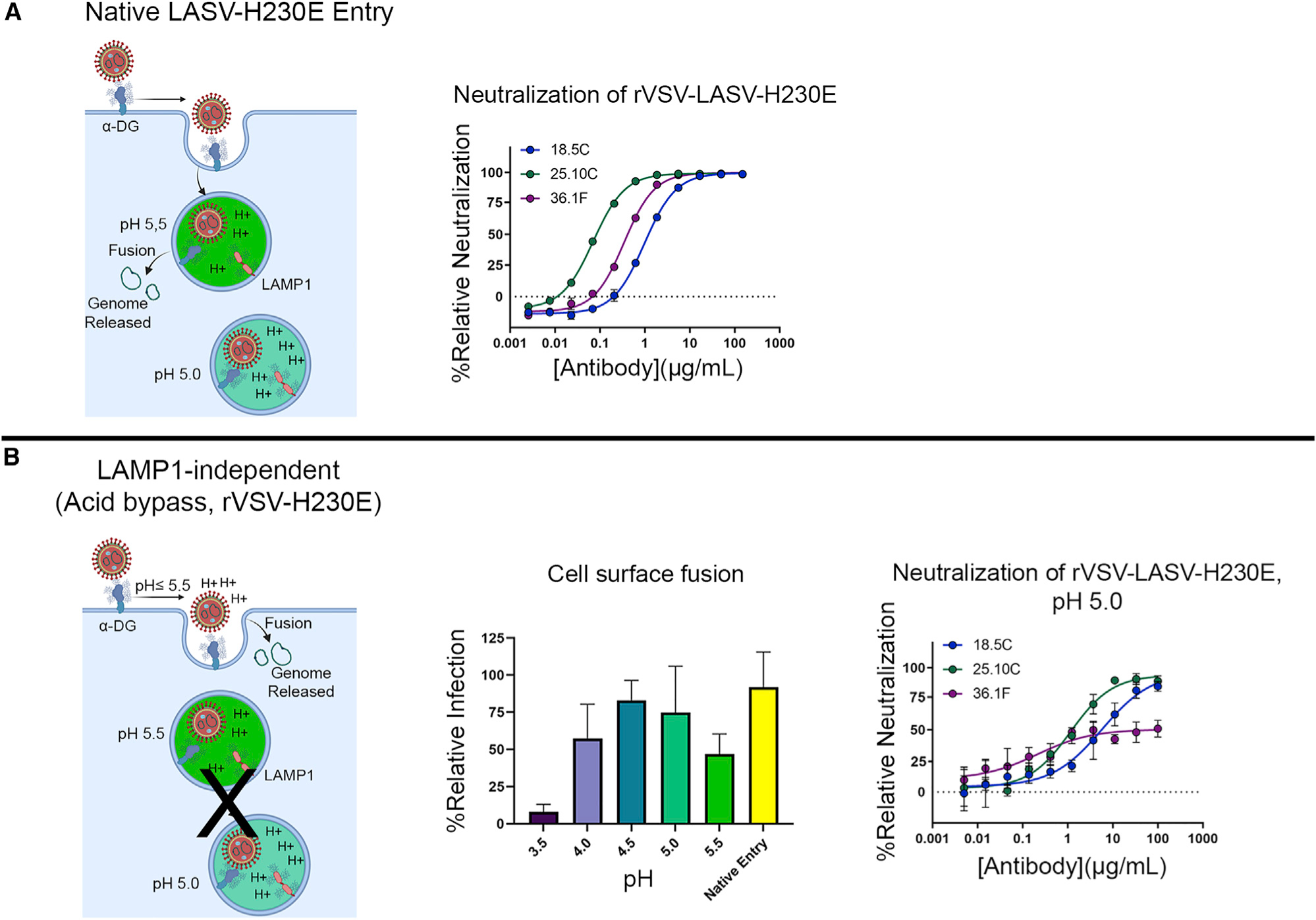
LAMP1 dependence of 36.1F neutralization (A) (Left) Schematic depicting the native entry pathway for rVSV-LASV-H230E. (Middle) Neutralization of rVSV-LASV-H230E by the indicated mAb under native entry conditions. (B) (Left) Schematic depicting entry of rVSV-LASV-H230E at the cell surface in wild-type Vero cells. In this entry pathway, viral fusion occurs in a LAMP1-independent manner. (Middle) The fusogenic profile of rVSV-LASV-H230E at the indicated pH is shown. Data were normalized to the total infectivity of virus under native entry conditions (i.e., endosomal dependent, and not exposed to either acid or lysosomotropic agents). Error bars indicate the mean± SD of two independent experiments, each performed in quadruplicate. (Right) Neutralization of rVSV-LASV-H230E by the indicated mAb at pH 5.0. For (A-middle and B-right), data are displayed as the relative neutralization as compared with a PBS control. Error bars indicate the SD of the mean of three independent experiments, each performed in duplicate.

A mutation in GP, H230E, has been described that allows LAMP1-independent cell-surface fusion at pH 5.5 instead of 4.5 (Cohen-Dvashi et al., 2016). Since mAb 36.1F does not interact with LASV pfGP at pH ≤ 4.5, we used H230E to evaluate the ability of 36.1F to neutralize in a LAMP1-independent manner. We found that even at pH 5.5, where 36.1F can bind GP, 36.1F cannot neutralize when LAMP1 is absent. Only 25.10C could effectively neutralize in a LAMP1-independent manner (Figure 6B, right).

Taken together, these results suggest that 25.10C and 36.1F have different neutralization mechanisms, despite their similar epitope. The lineage IV-specific 36.1F (with half maximal inhibitory concentration [IC50] of 4 nM [Robinson et al., 2016]), neutralizes by preventing association with LAMP1. It does not neutralize in the absence of LAMP1 and can only neutralize in earlier endosomal compartments before pH drops <4.5. In contrast, the pan-LASV 25.10C can neutralize by blocking GP association with LAMP1 and can also neutralize in an LAMP-1 independent, pH-independent manner, likely by blocking fusion of virus and host membranes through occupation of the GP2 fusion loop. As a result of neutralization even in the latest endosomal compartments, and likely of its dual mechanisms, 25.10C is more potent, with an IC50 of 0.1 nM.

## DISCUSSION

The two Fab-pfGP structures presented here demonstrate that antibodies in the GPC-A competition group bind to the side of the GP trimer, to an epitope that bridges GP1 and GP2 subunits, and which is ringed by five glycans, linked to Asn 79, 89, 99, and 224 in GP1 and Asn 365 in GP2. The GPC-A antibodies bind one Fab to one copy of GP, three Fabs per trimer. The antibodies arose from divergent germlines and converged on an overlapping quaternary epitope. A loop composed of residues 225 to 232 constitutes the longest sequential region contacted by both 25.10C and 36.1F. Protonation of H230 in this loop at pH < 6.0 is thought to trigger conformational adjustment of this region in GP1 to allow LAMP1 binding (Cohen-Dvashi et al., 2016; Li et al., 2016). Indeed, blocking of LAMP1 binding is a key mechanism of neutralization for both GPC-A antibodies.

Although their footprints overlap, the approach angles of the two antibodies are different: 25.10C binds at an orientation more parallel to the plane of the viral membrane while 36.1F binds down onto GP with its constant domains angled upward from the membrane. The different approach angles result in a modest, but impactful, shift in CDR H3 and light chain placement and a commensurate shift in the number of contacts with the GP2 subunit. The CDR H3 of 25.10C contacts GP just once through a salt bridge between mAb residue R101 and GP1 residue E76 and makes no contacts to GP2. The extensive network of contacts between GP2 and 25.10C is instead mediated solely by the light chain. In contrast, the CDR H3 of 36.1F forms six contacts with GP1, including E76, as well as several contacts to the H230 loop, but only a single contact to GP2. The slight variation in approach shifts the 36.1F light chain away from GP and results in an epitope that is ~95% GP1 and 5% GP2. The more even distribution of the 25.10C epitope across the GP1 and GP2 subunits (65% GP1 and 35% GP2) may allow 25.10C to also neutralize at a LAMP1-independent step, likely membrane fusion. In contrast, the 36.1F epitope, which is predominantly GP1, can only block LAMP1 receptor usage. Last, 36.1F shows marked reduction in interaction with GP at pH < 5 while 25.10C maintains robust binding even at pH 4. Hence, not only does 25.10C have a dual role in preventing viral infection, it can do so throughout the entry process. In contrast, likely 36.1F functions only in less acidic, early endosomal compartments.

Antibodies in the GPC-A competition group provide potent *in vitro* neutralization, and one of them, 25.10C achieves panLASV breadth. Yet, how these antibodies neutralize GP and what they recognize on GP was previously unknown. By characterizing the structural variation between the two GPC-A mAbs, we have identified highly antigenic features on LASV GP that provide potent antibody-mediated neutralization against the circulating LASV lineages. The structures and complementary analysis presented here demonstrate that GPC-A antibodies neutralize by preventing interaction with LAMP1, and also illuminate the differences between broadly reactive and lineage-specific recognition, less potent and more potent neutralization, and LAMP-dependent and LAMP1-independent neutralization at this site, information critical for much-needed therapies and vaccines.

### Limitations of the study

One limitation of the study is that both Fab-GP structures were solved using a stabilized, pre-fusion GP ectodomain and there may be subtle differences compared with antibody binding to the native, full-length GP on the surface of the virion. In addition, the rVSV-LASV-GP pseudovirus we used for neutralization assays may not completely recapitulate the characteristics of authentic LASV GP during infection. Neutralization assays using authentic viruses are needed to confirm our findings. Another limitation is that the exact molecular mechanism by which GPC-A antibodies block LAMP1 utilization by GP is unclear. LASV GP can bind LAMP1 only in acidic environments that induce conformational changes needed for the interaction. GPC-A antibodies could interfere with the GP-LAMP1 interaction either by preventing these acid-induced conformational changes in GP or by sterically occluding the LAMP1 epitope on GP. Further experiments are needed to determine the exact method by which GPC-A antibodies prevent the GP-LAMP1 interaction. In this study, we focused on the differences in neutralization mechanisms between 25.10C and 36.1F and the ability of 25.10C to block in both a LAMP1-dependent and -independent manner. Further mutagenesis and structural studies using GPs from various LASV lineages will provide further evidence for structural predictions regarding GPC-A antibody efficacy across circulating LASV lineages.

## STAR★METHODS

### RESOURCE AVAILABILITY

#### Lead contact

Correspondence and requests for materials should be addressed to K.M.H. (kmhastie@lji.org).

#### Materials availability

This study did not generate new unique reagents.

#### Data and code availability

Structure factors and associated model coordinates generated by X-ray crystallographic studies have been deposited at the protein DataBank (PDB: http://rcsb.org) under accession numbers PDB: 7S8H (Fab 36.1F bound structure) and PDB: 7S8G (Fab FNQI-25.10C) and are publicly available as of the date of publication. The atomic models and cryo-EM maps derived from the Fab 25.10C bound structure have been deposited at the PDB (PDB: http://rcsb.org) and in the Electron Microscopy Databank (EMDB: http://www.emdataresource.org/) under the following accession numbers PDB: 7TYV and EMD: EMD-2165 and are publicly available at the time of publication.

This paper does not report original code.

Any additional information required to reanalyze the data reported in this paper is available from the lead contact upon request.

### EXPERIMENTAL MODEL AND SUBJECT DETAILS

#### Cell lines

HEK293T (ATCC CRL-3216) and Vero (ATCC CCL-81) cells were cultured in high-glucose Dulbecco’s modified Eagle’s medium containing L-glutamine (DMEM, Invitrogen, Carlsbad, CA) supplemented with 10% fetal bovine serum (Omega Scientific, Tarzana, CA) and 1% penicillin-streptomycin solution (Penn/Strep). Cells were maintained at 37°C in a humidified atmosphere with 5% CO_2_. ExpiCHO cells were cultured in ExpiCHO expression medium and maintained at 37°C in a humidified atmosphere with 8% CO_2_. *Drosophila* S2 cells were cultured in Schneider’s *Drosophila* medium at 27°C in stationary flasks. Stable cell lines used for expression of proteins were adapted to serum free conditions in Insect Xpress media (Lonza) and maintained with shaking at 27°C.

#### Viruses

Recombinant VSV-ΔG-GFP virions were generated by pseudotyping with a synthetic, codon-optimized LASV GPC of LIV-Josiah.

### METHOD DETAILS

#### Generation of IgG

ExpiCHO-S cells were grown in shaker flasks in ExpiCHO expression medium in a humidified chamber at 37C, 8% CO2. Cells were passaged every three to four days achieving early log phase growth at 4–6 × 10^6^ cells/mL. One day prior to transfection, cells were diluted with pre-warmed ExpiCHO expression medium to a final density of 3–4 × 10^6^/mL. The day of transfection, cells were diluted back to 6 × 106/mL with ExpiCHO expression medium, in 25 mL final volume per flask. Cells were transfected with a 1 to 1.15 ratio of heavy chain to light chain plasmid DNA using the ExpiFectamine CHO Transfection Kit as per the manufacturer’s instructions. The following day, cells were fed by diluting 150 mL of Expi-CHO enhancer into 6 mL of ExpiCHO-Feed, and pipetting the entire mixture into the shaker flask of transfected cells. Cells were assayed for viability on Day 7 post transfection, and supernatant was collected when the viability dropped to 75%. Antibodies were purified from clarified supernatants via Protein A affinity chromatography using a HiTrap PrismA mAb select column. IgGs were eluted with 0.1 M Citrate pH 3.4, neutralized with a 1/10 volume of 1 M NaPhosphate, pH 8.0 and subsequently dialyzed into PBS.

#### Digestion and purification of IgG into fab fragments

Purified IgG was digested by incubating with 5% papain (w/w) for 3 h at 37°C. The resulting Fab fragments from both 25.10C and 36.1F were purified from Fc fragments using a Kappa select column (GE Healthcare) and further purified by size-exclusion chromatography (SEC) using an S200 Increase 10/300 column (GE Healthcare). Elbow mutations in 25.10C Fab were introduced using PCR primers containing the mutations. Subsequent cloning and Fab fragment preparation were performed as described above for WT 25.10C Fab.

#### Expression and purification of LASV GPCysR4 and derivatives

Expression and purification of the soluble LASV GPCysR4 ectodomain (pfGP) was performed as previously described (Hastie et al., 2017). Briefly, the LASV pfGP soluble ectodomain (residues 1–424) was modified to bear the dicysteine mutations R207C and G360C (cryoEM construct) or G243C and I350C (crystallography construct), the helix-breaking mutation E329P, and the mutations L258R and L259R to change the native S1P cleavage site to a furin protease cleavage site for production in *Drosophila* S2 cells (Invitrogen). S2 cells were grown to a density of 1 × 10^7^ cells per mL, and protein expression was induced using 500 μM CuSO_4_. Protein was purified from the supernatant by streptactin-affinity chromatography and the StrepII tags were removed by overnight incubation with EKMax (Thermo Fisher). The resulting protein was further purified by SEC using an S200 Increase or Superose 6 Increase column (GE Healthcare). The C terminus of the GPCysR4 cryo-EM construct (pfGP-TD) contains an added LPETG amino acid sequence at the C terminus that allows ligation to the trimerization domain (PDB: 1NOG).

#### Trimerization domain and sortase enzyme cloning and purification

The trimerization domain from *Thermoplasma acidophilum* (ATP:cobalamin adenosyltransferase; PDB: 1NOG) was cloned into the pET21a bacterial expression vector system (EMD-Millipore, formally Novagen) with an added penta-glycine motif and His6 tag at the N and C terminus, respectively. The recombinant protein was expressed in Rosetta2 *E. coli* cells (Invitrogen). Bacterial cells were cultured in LB broth at 37°C until the density reached an absorbance of 0.5–0.7 at 600 nm. Protein expression was then induced with 500uM IPTG (isopropyl-β-D-thiogalactopyranoside) and cells were grown overnight at 25C. Cultures were harvested via centrifugation and lysed in 50 mM Tris, pH 8.0, 300 mM NaCl, 30 mM imidazole using an M-100L Laboratory Microfluidizer (Microfluidics). Cellular debris was removed by centrifugation. Protein was purified from the supernatant by incubation with NiNTA-agarose beads (Qiagen). Bound proteins were eluted in 50 mM Tris pH 8, 300 mM NaCl and 300 mM imidazole and further purified over an S75 SEC column (GE Healthcare). Sample homogeneity was determined by SDS-PAGE.

SortaseA in the pET29 bacterial expression vector system was obtained from Addgene and contains a 6x-His tag at the C terminus (Addgene plasmid #75144; http://n2t.net/addgene:75,144; RRID:Addgene_75,144) (Chen et al., 2011). Recombinant protein was expressed in Rosetta2 E. coli cells (Invitrogen). Bacterial cells were cultured in LB broth at 37°C until the density reached an absorbance of 0.7 at 600 nm. Protein expression was induced using 500uM IPTG at 30°C for an additional 4 h. Cells were harvested by centrifugation and lysed using an M-100L Laboratory Microfluidizer (Microfluidics) in 50 mM Tris, pH 8.0, 300 mM NaCl, 30 mM imidazole and cellular debris was removed by centrifugation. The supernatant was incubated with NiNTA-agarose beads overnight at 4°C. Bound proteins were eluted using 200 mM imidazole, and further purified using an S75 SEC column.

#### LASV pfGP trimerization and purification

LASV pfGP-TD trimer was formed by ligating 40 μM LASV GPCysR4-LPETG monomer to 14 μM 1NOG trimerization domain using 1.35 μM SortaseA enzyme in a buffer containing 50 mM Tris pH7.5, 150 mM NaCl, and 2 mM CaCl_2_ for 1hr at room temperature. The ligation reaction was quenched with 50 mM iodoacetamide. The resulting pfGP-TD trimer was purified using an S200 Increase column.

#### Antibody-GP complex formation

Purified pfGP-TD was incubated with excess 25.10C Fab for at least 1hr at RT and Fab-pfGP-TD complexes were then purified by SEC using an S200 Increase column. Fractions corresponding to trimeric complexes were pooled and used for subsequent EM analysis. Ternary complexes of GP-Fab18.5C-Fab36.1F were produced by incubating a 1.5 molar excess of each Fab the pfGP monomers for at least one hour before separation on an S200 increase column. Fractions corresponding to trimeric GP-Fab complexes were pooled and used in crystallization trials.

#### X-Ray crystallographic structure determination and refinement

Crystal screening of 18.5C–36.1F-pfGP complexes and 25.10C-FNQI Fab fragments, each at 8–10 mg/mL, was carried out independently using sparse matrix screens and an Oryx crystallization robot (Douglas Instruments). LASV pfGP in complex with 18.5C and 36.1F Fabs formed thin crystal plates in 0.1 M Tris, pH 8.0, 1.25 M LiCl and 15% PEG 8k. Crystals were cryo-protected in mother liquor with 10% glycerol. The 25.10C-FNQI Fab formed hexagonal crystals in 0.1 M MES/sodium hydroxide pH 6.0, 40% (v/v) PEG 400, and 5% (w/v) PEG 3000. All crystals were flash frozen in liquid nitrogen.

X-Ray diffraction data for both the 18.5C-36.1F-pfGP complex and the 25.10C-FNQI Fab were collected on APS beamline 23-ID-D with data extending to 2.9Å and 2.0 Å, respectively. Data for 18.5C-36.1F-GP were processed using XDS (Kabsch, 2010) and were indexed in space group P32_1_ with one ternary complex in the asymmetric unit. Molecular replacement was performed using a pfGP monomer (PDB code 5VK2).

For the 25.10C-FNQI Fab structure, crystals diffracted to 2.6Å. Data integration and scaling were performed using the autoPROC implementation of XDS and AIMLESS (Vonrhein et al., 2011). Crystals were indexed in the P12_1_1 space group and isotropic data were used for model building and refinement. Data quality was assessed using the CCP4 implementation of CTRUNCATE (PMID: 21460441). Twin fraction estimates by twinning operators (H-test, Britton plot, and ML-Britton test) indicated the data was approximately 27% pseudo-merohedral twinned. Molecular replacement was carried out in PHENIX (Afonine et al., 2018) using another arenavirus mAb structure (unpublished) with the CDRs removed as the search model.

For both structures, model building, and manual verification of Ramachandran values and rotamers was carried out using Coot (Casañal et al., 2020), followed by refinement of the models in PHENIX (Afonine et al., 2018). For the 18.5C-36.1F-pfGP model, Privateer (Agirre et al., 2015) and PDBcare (Lütteke and von der Lieth, 2004) were used to check discrepancies in glycan connectivity, nomenclature and glycosidic torsion angles. The twin operator (-h, -k, h + l) was applied in the final round of refinement of the 25.10C-FNQI Fab structure, reducing the Rwork by ~5%. Map to model quality was performed using MolProbity (Chen et al., 2010; Williams et al., 2018) to validate the quality of the final models (Tables S1 and S2). PyMOL was used for visualization of final atomic models (Schrodinger, 2010). The 25.10C-FNQI Fab structure includes residues 3–217 of the heavy chain with a disordered region from 134 to 140, and residues of 1–209 of the light chain. The final 18.5C-36.1F-pfGP model contains residues 59–422 of pfGP monomer, with disordered regions from 171 to 179, residue 206, and 256–259. The 18.5C Fab model contains residues 1–224 of the heavy chain and 1–214 of the light chain, while the 36.1F Fab contains residues 1–226 of the heavy chain and 1–216 of the light chain.

#### Cryo-EM sample preparation and data collection parameters

Purified 25.10C-pfGP-TD trimer was diluted to a working concentration of 0.8 mg/mL in a buffer containing 50 mM Tris pH 7.5 and 150 mM NaCl. Then, 3 μL protein was mixed with 1 μL LMNG detergent (0.02 mM), and 3 μL of this mixture was adsorbed on a Quantifoil 2/1 grid (QUANTIFOIL) that was previously plasma-cleaned for 30 s in a NanoClean Model 1070 (Fischione Instruments) with a ratio of 25% oxygen to 75% argon. Excess protein solution was blotted for 10 s before immediate plunge freezing into liquid ethane using an FEI Vitrobot (Thermo Fisher Scientific). Frozen grids were imaged using a Titan Krios (Thermo Fisher Scientific) equipped with a Gatan K2 detector and operated in super-resolution mode. Movies were collected using SerialEM (Mastronarde, 2005) at a magnification of 463,003× at the detector that corresponds to a calibrated pixel size of 0.548 Å/pixel. Micrographs were collected in a single session with a defocus range between 1.0 and 2.5 micron underfocus.

#### Cryo-EM data processing

All data processing was carried out using cryoSPARC v2.14.2 (Punjani et al., 2017). Cryo-EM movies were motion-corrected and averaged to a single frame using Patch motion correction. CTF estimation was performed using Patch CTF estimation. Particle projections were picked from the micrographs using the CryoSPARC blob picker, then downsampled by a factor of 2, and subjected to reference-free 2-D class averaging to yield a final particle stack of 1,150,349 particle projections that were used for all ab-initio reconstructions and 3-D refinements. The final C3 symmetrized reconstruction yielded a resolution of 3.0Å (Table S3). Local resolution estimates were performed using cryoSPARC’s built-in implementation, and post-processing of the cryo-EM reconstruction was performed using DeepEMhancer (Sanchez-Garcia et al., 2020). The angular distribution of particle projections in the final cryo-EM reconstruction was calculated using PyEM (Asarnow et al., 2019), and visualization of reconstructions performed using ChimeraX (Pettersen et al., 2021).

#### Atomic model building into Cryo-EM reconstruction

The 25.10C-pfGP atomic model was modeled from the pfGP portion of the 36.1F crystal structure, while the 25.10C heavy and light chains were modeled from the 25.10C-FNQI crystal structure. GP and Fab models were first docked into the cryo-EM reconstruction using ChimeraX (Pettersen et al., 2021) and then subjected to real-space refinement in PHENIX (Afonine et al., 2018). The initial mapmodel FSC calculated in Phenix indicated a fit of 3.5Å. To avoid the possibility of overfitting, the final cryo-EM reconstruction was lowpass filtered to 3.5Å using *relion_image_handler* (Scheres, 2012), and the map-model FSC was recalculated after another refinement step in PHENIX. Rotamer and Ramachandran outliers were manually adjusted using Coot (Casañal et al., 2020). PyMOL was used for visualization of final atomic models (Schrodinger, 2010).

#### Recombinant expression of LAMP1-RbFc

LAMP1 (residues 29–351) used in blocking ELISAs was produced as a Rabbit-Fc fusion protein as previously described (Hastie et al., 2019). Briefly, LAMP1-RbFc was transiently expressed in CHO-Flpln cells stably expressing the β-galactoside α−2,3-sialyltransferase ST3GAL4. LAMP1-Fc was purified from the supernatant of transfected cells by Protein A affinity chromatography. Bound proteins were eluted with 0.1mM glycine, neutralized with a 1/10 volume of 1 M Tris pH 8 and subsequently dialyzed into 25 mM sodium citrate buffer pH 5.0 with 150 mM NaCl.

#### ELISA analysis of LAMP1 to soluble GP trimers

ELISAs were performed as previously described (Hastie et al., 2017, 2019). Briefly, ELISA plates (Corning, Kennebunk, ME) were coated with 2.5 μg/mL streptavidin in PBS for 3 h at RT, and blocked with 3% BSA in PBS for 2 h at RT. LASV pfGP-TD (1 μg/mL) either alone or in complex with mAb (10 μg/mL) was diluted in PBS for 1hr at RT before coating on ELISA plates (Corning, Kennebunk, ME). After blocking, a dilution series of LAMP1-RbFc starting at 10 μg/mL was bound to coated protein for 2 h at RT in 50 mM citrate pH 5.0 with 150 mM NaCl buffer. Wells were washed extensively and bound LAMP1-RbFc was detected using goat anti-rabbit-IgG(H + L), mouse/human ads-HRP antibody (Southern Biotech, 1:5,500) for 1 h at RT.

#### Generation of LASV GP plasmids and pseudoviruses

Recombinant VSV-ΔG-GFP pseudovirions bearing various LASV lineages and mutants of LASV GP were generated by transfecting 293T cells with phCMV3 expressing the indicated version of LASV GP using TransIT LT1 (Mirus, Madison, WI) per the manufacturer’s instructions. 24 h post-transfection, the medium was removed and cells were infected with rVSV-G pseudotyped ΔG-GFP parent virus (VSV-G*ΔG-GFP) at an MOI of 3 for 2 h with rocking. Virus was removed, cells were washed two times with OPTI-MEM containing 2% FBS (OPTI-2) and fresh OPTI-2 was added to cells. Supernatants containing rVSV-LASV were removed from cells 16 h post-infection and clarified by centrifugation. Titers were quantified as the number of fluorescent forming units (ffu/mL) using a CellInsight CX5 imager and automated enumeration of cells expressing GFP. Aliquots of virus were flash frozen in liquid nitrogen and stored at −80°C.

#### Neutralization assays

Vero cells were seeded in 96-well plates (Corning Life Sciences) at a cell density of 20,000 cells/well in DMEM with 10% FBS and 1% Penn/Strep 5 h prior to infection. Antibodies were diluted to f 0.3 mg/mL (100 nM) and further diluted in a 10-fold dilution series. Each Ab dilution was then mixed 1:1 with a pre-titrated amount of rVSV-LASV-GP and incubated for 1 h at 37°C. Media was removed from the cell monolayer and the Ab/virus mixture was added to duplicate wells and incubated overnight in 5% CO_2_ at 37°C. Cells were fixed using 4% paraformaldehyde (PFA; Electron Microscopy Sciences) in PBS, and cell nuclei were stained using 1 μg/mL Hoechst-33342. Cells were imaged using a CellInsight CX5 imager (Thermo Fisher) and infection was quantitated by automated enumeration of cells expressing GFP.

#### Retroviral transduction of vero cells with LAMP1D384

Human LAMP1Δ384 was cloned into the lentiviral expression vector pCW62. The LAMP1 Δ384 retroviral vector was transfected using TransIT transfection reagent into a confluent monolayer of HEK-293T cells together with the plasmids pCAGGS-VSV-G and pSPAX2, and incubated in 5% CO_2_ at 37°C for 48 h. Virus was harvested from the supernatant at 48 h post-transfection, filtered and applied to Vero cells. Transduced Vero cells were selected with blasticidin S HCl (ThermoFisher) and resistant cells were pooled as a polyclonal cell line.

#### Immunofluorescence of LAMP1Δ384 transduced veros and non-transduced veros

Vero or Vero-LAMP1Δ384 cells were seeded at 250,000 cells/well in 24-well plates (Corning ref 3524). Cells were washed with PBS and fixed using 4% PFA for 20 min at RT. Wells were blocked with PBS containing 1% BSA for 1 h at RT. A 1:500 dilution of mouse anti-human CD107a (LAMP1; Southern Biotech) was added to transduced and non-transduced cells, which were washed with PBS with 5% Tween 20 (PBST) and incubated for 1 h at RT with AlexaFluor 488-conjugated goat anti-mouse IgG (Thermo Fisher) diluted 1:200. After a final wash with PBST and PBS, cell nuclei were stained using Hoechst-33342 and imaged on a CellInsight CX5.

#### Acid bypass neutralization and fusion assays

For all experiments, Vero cells were seeded in 96-well plates (Corning 3603) at a density of 20,000 cells/well. Antibodies were mixed with diluted rVSV-LASV-GP viruses (35,000 ffu) and incubated for 1 h at 37°C. All samples were diluted into OPTI-2.

For neutralization experiments, the Ab/virus mixture was added to pre-chilled Vero cells and bound to the cell surface by centrifugation (2,500 rpm) for 1 h at 4°C. Cells were placed on ice and washed with cold PBS. To induce fusion, cells were incubated with pre-warmed 50 mM citrate buffer pH 4.0 or pH 5.0 with 150 mM NaCl or PBS (pH 7.5) for 5 min at 37°C. Cells were placed back on ice and endosomal fusion of pH adjusted cells was halted by the addition of cold OPTI-2 containing 20 mM NH_4_Cl. Control wells were washed with cold OPTI-2. All cells were then incubated overnight in warm OPTI-2 (20 mM NH_4_Cl for pH adjusted cells and OPTI-2 for control wells) in 5% CO_2_ at 37°C. All measurements were performed in duplicate and data are displayed as the relative neutralization as compared to a PBS control. Cells were fixed, stained, imaged, and GFP counts enumerated on a CellInsight CX5 as previously described.

Membrane fusion assays using rVSV-LASV-GP were performed using both wildtype Vero cells and LAMP1Δ384-Vero cells. Experiments were carried out as described above, except without antibody present. After washing with cold PBS, pre-warmed citrate buffers ranging from pH 3.5–5.5 (50 mM Citrate/150 mM NaCl) were used to induce fusion. Cells were incubated with citrate buffers for 5 min at 37°C before placing cells back on ice and quenching with cold OPTI-2 containing 20 mM NH_4_Cl (cold OPTI-2 was used for control wells). All cells were then incubated overnight in warm OPTI-2 (20 mM NH_4_Cl for pH adjusted cells and OPTI-2 for control wells) in 5% CO_2_ at 37°C. All measurements were performed in quadruplicate and data were normalized to the total infectivity of virus under native entry conditions (i.e., endosomal dependent, and not exposed to either acid or lysosomotropic agents). Cells were fixed, stained, imaged, and GFP counts enumerated on a CellInsight CX5 as previously described.

#### ELISA of GPC-A mAb-LASV GP interactions at neutral and acidic pH

Half-area ELISA plates (Corning, Kennebunk, ME) were coated with 2.5 μg/mL Streptavidin in PBS for 2–3 h at RT, and blocked with 3% milk in PBS for 1 h at RT. After blocking, 1 μg/mL LIV LASV pfGP-TD was captured for 2 h at RT, followed by incubation with the indicated antibody concentration overnight at 4°C. The antibody GP solution was removed and plates were washed three times using PBS with 0.05% Tween 20, followed by treatment with PBS or 50 mM citrate/150 mM NaCl buffer adjusted to the indicated pH value for 1 h at RT. The plates were then washed three times with PBS-Tween20. Detection was performed using horseradish peroxidaseconjugated goat α-human IgG antibody reagent (KPL) for 1 h at RT. After an additional wash step, color was developed by the addition of 3,3,′5,5′ tetramethylbenzidine (TMB)-H_2_O_2_ (Thermo Fisher) for 5 min at RT. Development was stopped by the addition of 2N sulfuric acid. Color was read as absorbance (optical density) at 450nm.

#### Generation of LASV lineage alignment

The amino acid sequences of GP from LASV lineage I (Pinneo), LASV lineage II (LASV-237-Nig2010), lineage III (Nig08-A18), lineage V (Soromba-R), lineage VI (KAK-428), and lineage VII (Lassa/H.sapiens-tc/TGO/2016/812939), were aligned to the sequence of the GP from lineage IV (Josiah strain) using Clustal Omega in Jalview 2.11.1.4 (Waterhouse et al., 2009) with manual notations.

### QUANTIFICATION AND STATISTICAL ANALYSIS

Statistical details of experiments, including numbers of replicates and measures of precision (standard deviation, SD) can be found in the figure legends, figures, and results and methods. Does-response neutralization curves were fit to a logistic equation by non-linear regression analysis. All analyses were performed with GraphPad Prism, version 9.1.0.

## Supplementary Material

1

## Figures and Tables

**KEY RESOURCES TABLE:** 

REAGENT or RESOURCE	SOURCE	IDENTIFIER

Antibodies		

Goat Anti-Mouse IgG (H + L) XAbs-Alexa Fluor 488	Invitrogen	Cat# A-11001; RRID: AB_2534069
Goat Anti-Human IgG-HRP	Invitrogen	Cat# A18814; RRID: AB_2535591
Purified Human IgG	Invitrogen	Cat# 02–7102; RRID: AB_2532958
Rabbit-IgG/mouse-human XAbs	Southern Biotech	Cat# 4050–05; RRID: AB_2795955
Mouse anti-Human CD107a-UNLB (LAMP1)	Southern Biotech	Cat# 9835–01; RRID: AB_2797102
18.5C	Robinson, et al., 2016	N/A
25.10C	Robinson, et al., 2016	N/A
36.1F	Robinson, et al., 2016	N/A
25.10C elbow mutant	This study	N/A

Bacterial and virus strains		

VSV-ΔG-GFP	Karafast	Cat# EH1020
rVSV-LASV LIV (Josiah)	This study	N/A

Chemicals, peptides, and recombinant proteins		

EKMax	Invitrogen	Cat# E180-01
Papain	Sigma	Cat# P3125
ExpiCHO expression medium	Thermo Fisher Scientific	Cat# A2910001
Normal goat serum	Novex	Cat# PCN5000
BioLock	Iba	Cat# 2-0205-250
Blasticidin S HCl	Thermo Fisher Scientific	Cat# A1113903
PFA	Electron Microscopy Sciences	Cat# 15710
Hoechst	Thermo Fisher Scientific	Cat# 62249
TransIT-LT1	Mirus	Cat # MIR 2304
Effectene	Qiagen	Cat# 301425
LASV LIV GPCysR4	Hastie, et al., 2017	N/A
LAMP1-RbFc	Hastie, et al., 2017	N/A
Lauryl maltose neopentyl glycol	Anatrace	NG310

Critical commercial assays		

TMB substrate kit	Peirce	Cat# 34021
ExpiFectamine CHO Transfection kit	ThermoFisher	Cat# A29129

Deposited data		

Structure of LASV GPC bound to Fabs 18.5C and 36.1F	Protein Data Bank (rcsb.org)	PDB: 7S8H
Structure of LASV GPC bound to Fab 25.10C	Protein Data Bank (rcsb.org) Electron Microscopy Data Bank (http://www.emdataresource.org/)	PDB: 7TYV EMD: EMD-2165
Structure of Fab 25.10C-FNQI	Protein Data Bank (rcsb.org)	PDB: 7S8G

Experimental models: Cell lines		

293T	ATCC	CRL-3216
Vero	ATCC	CCL-81
ExpiCHO-S	Thermo Fisher Scientific	Cat# A29127
Flp-In CHO	Thermo Fisher Scientific	Cat# R75807
Drosophlia S2	Thermo Fisher Scientific	Cat# R69007

Recombinant DNA		

Empty vector: phCMV3	Genlantis	Cat# P003300
Empty vector: pMTpuro	Addgene	Addgene #17923
pCAGGS-VSV-G	Kerfast	Cat# EH1017
phCMV3-LASV LIV-Josiah	Hastie, et al., 2017	N/A
phCMV3-LASV LIV-Josiah H230E	This study	N/A
pMTpuro-LASV GPCysR4	Hastie, et al., 2017	N/A
pcDNA3.1-LAMP1	Addgene	Cat# 45147
pcDNA3.1-LAMP1-Δ384	This study	N/A
pCW62	Harvard Plasmid Repository	#EvNO00438621
pSPAX2	Addgene	Cat# 12260

Software and algorithms		

XDS	Kabsch (2010)	http://xds.mpimf-heidelberg.mpg.de
Phenix, versions 1.19	Afonine et al., (2018)	https://www.phenix-online.org
Coot	Casañal et al., (2020)	https://www2.mrc-lmb.cam.ac.uk/personal/pemsley/coot/
Molprobity	Chen et al. (2010)	http://molprobity.biochem.duke.edu
PDB Care	Lütteke and von der Lieth (2004)	http://www.glycosciences.de/tools/pdb-care/
Privateer	Agirre et al. (2015)	CCP4i package
Pymol Molecular Graphics System	Schrodinger (2010)	https://pymol.org
GraphPad Prism 9	GraphPad Software, Inc	https://www.graphpad.com
SerialEM	Mastronarde (2005)	https://bio3d.colorado.edu/SerialEM/
cryoSPARC	Punjani et al., (2017)	https://cryosparc.com/
RELION	Scheres (2012)	https://github.com/3dem/relion
UCSF ChimeraX	Pettersen et al., (2021)	https://www.cgl.ucsf.edu/chimerax/
DeepEMhancer	Sanchez-Garcia et al. (2020)	https://github.com/rsanchezgarc/deepEMhancer
UCSF PyEM	Asarnow et al., (2019)	https://github.com/asarnow/pyem
Jalview	Waterhouse et al., (2009)	https://www.jalview.org/

Other		

StrepTrap HP	GE Healthcare	Cat# 28907547
HiTrap MabSelect PrismA	GE Healthcare	Cat# 17549851
HiTrap KappaFabSelect	GE Healthcare	Cat# 17545811
Superdex 75 Increase 10/300 GL	GE Healthcare	Cat# 29148721
Superdex 200 Increase 10/300 GL	GE Healthcare	Cat# 28990944
Superose 6 Increase 10/300 GL	GE Healthcare	Cat# 29091596
Oryx Crystallization Robot	Douglas Instruments	https://www.douglas.co.uk
CellInsight CX5 High Content Screening Platform	Thermo Fisher Scientific	Cat# CX51110
Titan Krios	Thermo Fisher Scientific	https://www.thermofisher.com/us/en/home.html
Holey Carbon C-flat 2/1 400 mesh copper grids	Electron Microscopy Sciences	https://www.emsdiasum.com/
PELCO easiGlow Glow Discharge Cleaning System	Ted Pella	https://www.tedpella.com/easiGlow_html/Glow-Discharge-Cleaning-System.aspx
Vitrobot Mark IV	Thermo Fisher Scientific	https://www.thermofisher.com/us/en/home/electron-microscopy/products/sample-preparation-equipment-em/vitrobot-system.html

## Highlights

- Lassa virus glycoprotein structures bound to GPC-A mAbs reveal lineage specificity
- Pan-Lassa virus mAb 25.10C binds a quaternary epitope spanning GP1 and GP2 subunits
- GPC-A mAbs 25.10C and 36.1F block LAMP1 binding to the Lassa virus glycoprotein
- mAb 25.10C neutralizes Lassa virus at endosomal pH while 36.1F does not
