# Supplementary material for: Delineating the mechanism of anti-Lassa virus GPC-A neutralizing antibodies
Source: Cell Rep. Author manuscript; Available in PMC 2022 Jul 6. (PMC9258627; doi:10.1016/j.celrep.2022.110841)
Supplement: 1 [file NIHMS1810671-supplement-1.pdf]

Cell Reports, Volume *39*

## Supplemental information

# Delineating the mechanism of anti-Lassa virus GPC-A neutralizing antibodies

**Adrian S. Enriquez, Tierra K. Buck, Haoyang Li, Michael J. Norris, Alex Moon-Walker, Michelle A. Zandonatti, Stephanie S. Harkins, James E. Robinson, Luis M. Branco, Robert F. Garry, Erica Ollmann Saphire, and Kathryn M. Hastie**

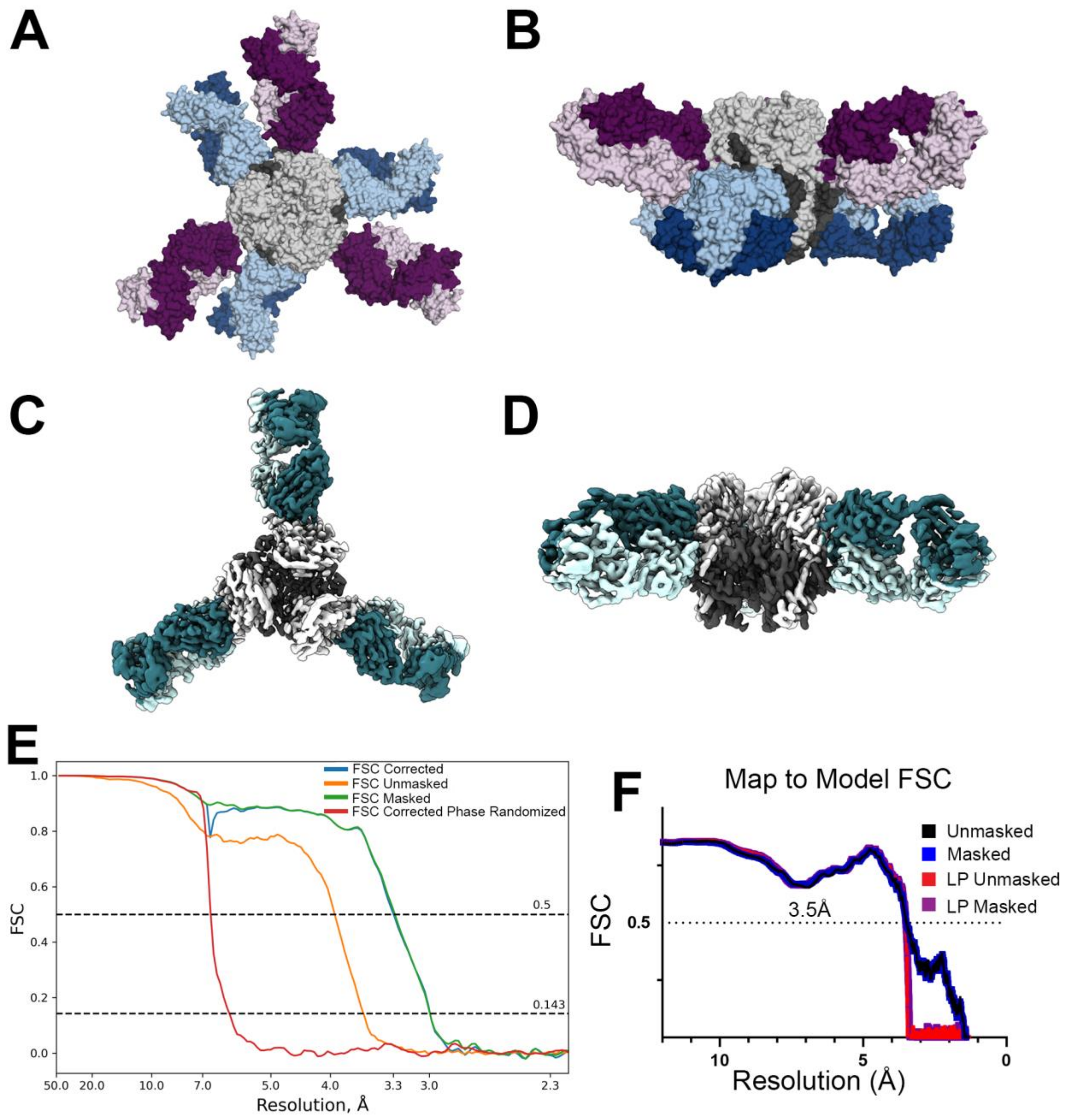


**Figure S1. Structure determination of GPC-A Fab-pfGP complexes, related to Figure 1. (A)** Surface view representation of the 18.5C-36.1F-pfGP ternary complex along the trimeric axis. **(B)** An orthogonal side-view of the ternary complex seen in “A”. **(C)** Top-view along the trimeric axis of the 25.10C-pfGP-TD cryo-EM reconstruction. **(D)** Side-view of the 25.10C-pfGP-TD complex seen in “C”. **(E)** Fourier shell correlation (FSC) depicting the global resolution of the final 25.10C-pfGP-TD cryo-EM reconstruction with 3-fold rotational symmetry applied. **(F)** FSC between the sharpened cryo-EM reconstruction and atomic model indicates a map-model correlation up to 3.5Å. To eliminate the possibility of overfitting, the map was low pass filtered to 3.5Å using *relion_image_handler* and the map-model FSC was recalculated after the final refinement step.

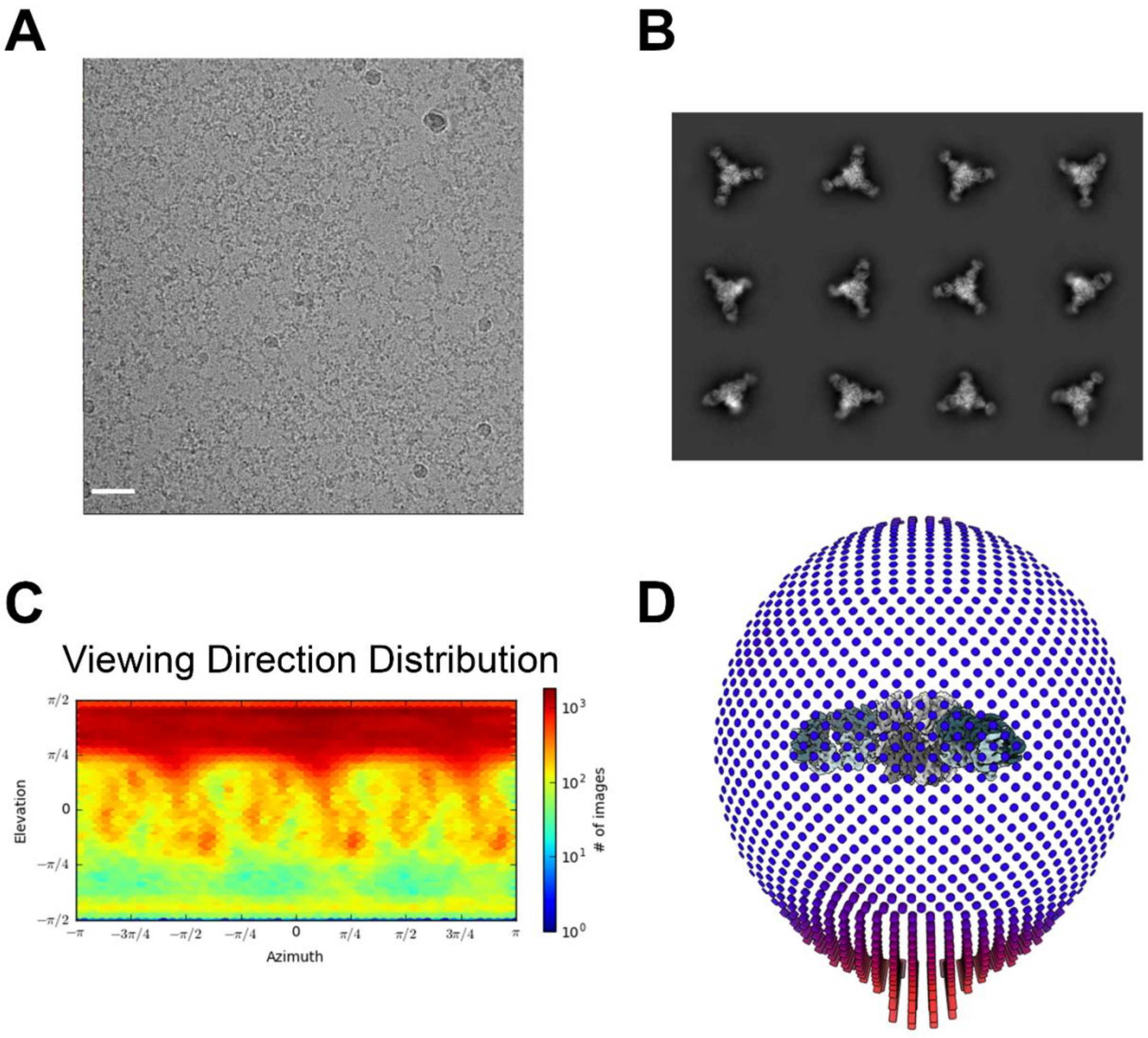


**Figure S2. Data-processing and validation of the 25.10C-pfGP-TD cryo-EM reconstruction, related to Figure 1. (A)** Representative micrograph of the 25.10C-pfGP-TD cryo-EM data-set. Scale bar: 20nm. **(B)** Representative reference-free 2-D class averages. **(C)** Viewing direction distribution histogram depicting the diversity of particle projection views present in the data set. **(D)** 3-D representation of the viewing direction distribution of the particle projections used for the final cryo-EM reconstruction. Particle projections contributing to the reconstruction are represented as bars with the height and color proportional to the number of projections in the indicated orientation.

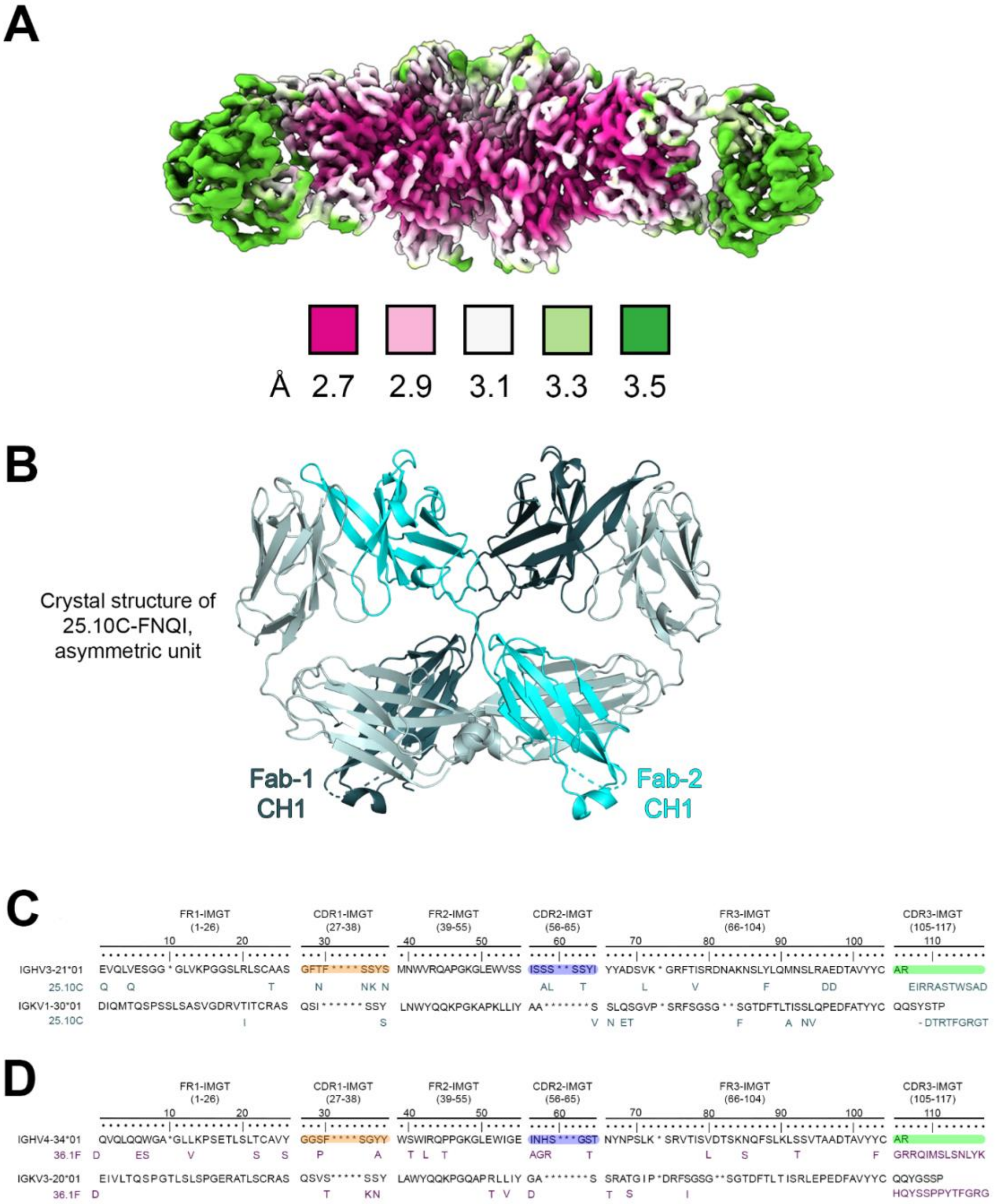


**Figure S3. The 25.10C-pfGP-TD complex is conformationally labile, related to Figures 1 and 2. (A)** Local resolution estimates of the 25.10C-pfGP-TD cryo-EM reconstruction colored as a heat map with pink and green corresponding to high and lower resolution density, respectively. The values for the resolution gradient were chosen to optimally illustrate the conformational flexibility within the 25.10C-pfGP-TD complex. **(B)** Atomic model of the 25.10C-FNQI Fab domain-swapped dimer. **(C)** Sequence alignment of the complementarity determining regions (CDR) of the 25.10C VH and VL domains with their germline progenitor. **(D)** Sequence alignment of the CDRs of the 36.1F VH and VL domains with their germlines. Sequences are numbered based on the IMGT numbering scheme. Residues that vary from the germline are colored in green (25.10C) and purple (36.1F).

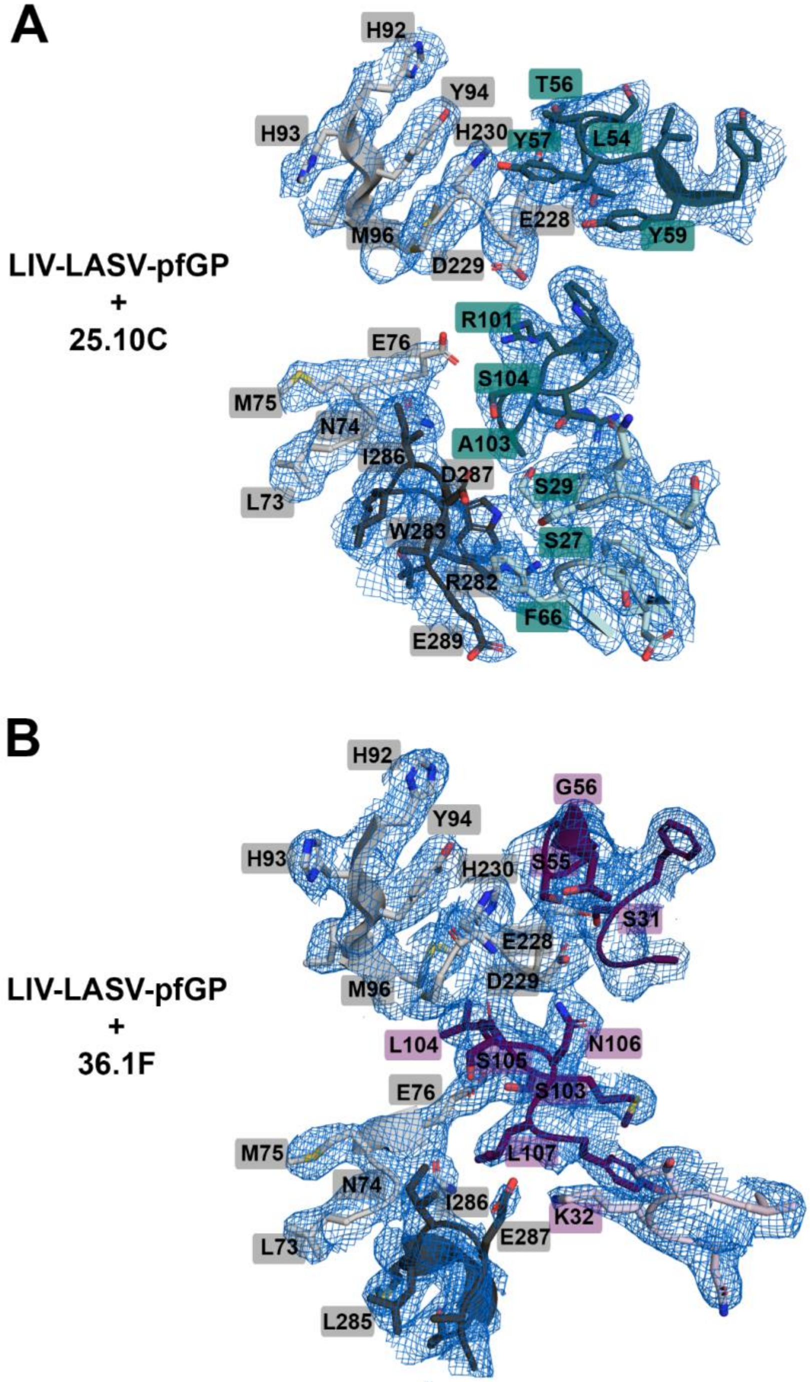


**Figure S4. Density maps with fitted atomic models for the 25.10C and 36.1F Fab-GP structures, related to Figure 2. (A)** Cryo-EM density map (mesh representation) for 25.10C Fab-LASV pfGP contacts with pfGP residues highlighted in gray and 25.10C residues in green. The cryo-EM map is shown at an isosurface threshold value of 0.1. **(B)** X-ray crystallography density map (mesh representation) for 36.1F Fab-LASV pfGP contacts with pfGP residues colored in gray and 36.1F in purple. The X-ray crystallography map is contoured at 1σ.

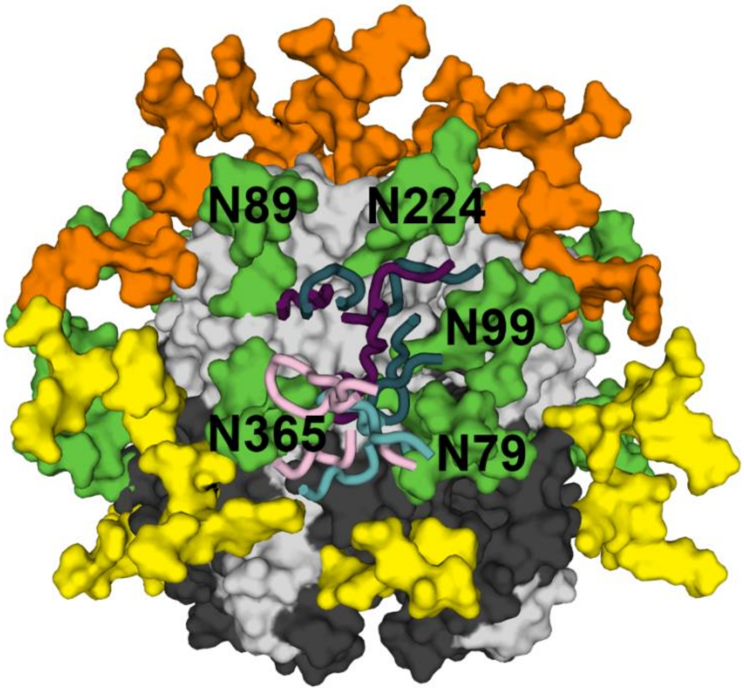


**Figure S5. GPC-A antibodies target a quaternary epitope nestled between glycans in GP1 and GP2, related to Figure 2.** Model of the fully glycosylated LASV pfGP trimer (adapted and modified from Watanabe *et al.*, 2018), illustrating that the GPC-A epitope is nestled between glycans N79, N89, N99, and N224 in GP1, and N365 in GP2. The heavy and light chain CDRs for 25.10C and 36.1F are colored dark and light green and pink, respectively. The aforementioned glycans that surround the GPC-A epitope are colored green, while glycans in GP1 and GP2 distant from the GPC-A epitope are colored orange and yellow, respectively.

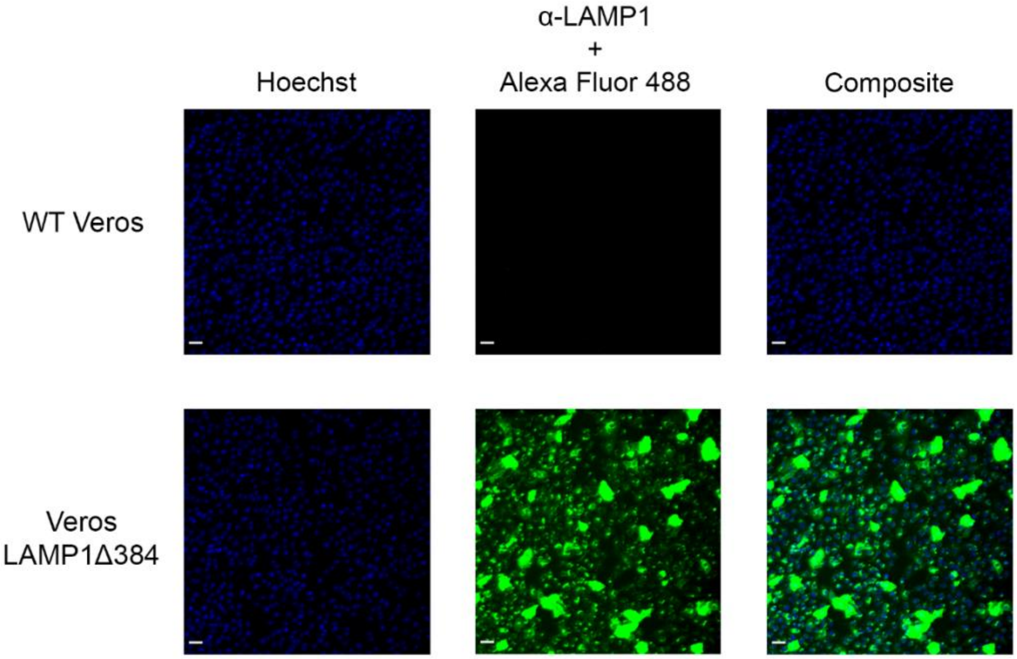


**Figure S6. Cell Surface Expressed LAMP1, related to Figure 5.** Wild-type Vero cells and Vero cells transduced with lentivirus expressing mutant LAMP1Δ384 were stained with Hoechst 33342 (nuclei) and probed for cell surface LAMP1 using an α-LAMP1 primary antibody and a secondary antibody conjugated to Alexa Fluor 488 dye (green). Scale bar: 50 μm.

**Table S1. LASV pfGP in complex with Fabs 18.5C and 36.1F. Related to Figure 1.**

| PDB Accession Code | 7S8H |
|---|---|
| **Data Collection** | |
| Space group | P321 |
| Cell Dimensions | |
| a, b, c (Å) | 163.058, 163.058, 173.548 |
| α, β, γ (°) | 90, 90, 120 |
| Resolution (Å) | 47.18-2.7 (2.797-2.7) |
| Total Reflections | 735054 |
| Unique Reflections | 73503 |
| Multiplicity | 10 |
| $R_{pim}$ | 0.07952 (1.189) |
| I / σ (I) | 9.53 (0.60) |
| $CC_{1/2}$ | 0.996 (0.26) |
| Completeness (%) | 99.92 (99.81) |
| | |
| **Refinement** | |
| Resolution (Å) | 47.18-2.7 (2.797-2.7) |
| No. reflections | 73476 (7253) |
| $R_{work}/R_{free}$ | 21.75/25.08 |
| | |
| No. atoms | 9824 |
| Protein | 9376 |
| Ligand/ion | 384 |
| B-factors | 79.91 |
| Protein | 78.72 |
| Ligand/ion | 112.10 |
| R.m.s deviations | |
| Bond lengths (Å) | 0.005 |
| Bond angles (°) | 0.77 |
| Ramachandran plot | |
| % favored | 97.32 |
| % allowed | 2.68 |
| % outliers | 0.00 |
| Molprobity | |
| Clashscore | 6.08 |

*Values in parentheses are for the highest-resolution shell.

**Table S2. 25.10C-pfGP-TD cryo-EM data collection/processing. Related to Figure 1.**

| | | |
|---|---|---|
| EMDB Identifier Code | EMD-26195 | |
| PDB Accession Code | 7TYV | |
| **Data Collection** | | |
| Microscope | Titan Krios | |
| Voltage (kV) | 300 | |
| Detector | Gatan K2 Summit | |
| Defocus range (µm) | 1.0-2.5 | |
| Magnification | 46,300X | |
| Movies | 2879 | |
| frames per movie | 40 | |
| Exposure timer per frame (ms) | 100 | |
| Dose rate (e-/pixel/sec) | 10 | |
| Total dose per movie (e-/Å$^2$) | 55 | |
| Movie micrograph pixel size (Å/pixel) | 0.548 | |
| Number of particles in final reconstruction | 1,150,349 | |
| Symmetry applied | C3 | |
| Map resolution (Å) (FSC=0.143) | 3.00 | |
| Map resolution (Å) (FSC=0.5) | 3.28 | |
| | | |
| **Model Statistics** | | |
| Chains | 12 | |
| Atoms | 17306 | |
| Residues (protein) | 2114 | |
| Water | 0 | |
| Ligands | BMA: 7, NAG: 37, MAN: 14 | |
| **Map to Model** | Masked | Unmasked |
| FSC 0/0.143/0.5 | 3.4/3.4/3.5 | 3.5/3.5/3.5 |
| CC* (mask) | 0.79 | |
| CC (box) | 0.82 | |
| CC (peaks) | 0.77 | |
| CC (volume) | 0.78 | |
| Mean CC for ligands | 0.75 | |
| R.m.s deviations | | |
| Bond lengths (Å) | 0.003 | |
| Bond angles (°) | 0.583 | |
| Ramachandran plot | | |
| % favored | 95.67 | |
| % allowed | 4.33 | |
| % outliers | 0.00 | |
| Molprobity | | |
| Clashscore | 7.47 | |
| EM-Ringer | 2.00 | |

*Cross correlation

**Table S3. Fab 25.10C-FNQI. Related to Figure 1.**

| PDB Accession Code | 7S8G |
|---|---|
| **Data Collection** | |
| Space group | P1211 |
| Cell Dimensions | |
| a, b, c (Å) | 71.6, 72.718, 90.014 |
| α, β, γ (°) | 90, 113.27, 90 |
| Resolution (Å) | 65.777-2.566 (2.610-2.566) |
| Total Reflections | 89354 |
| Unique Reflections | 27076 |
| Multiplicity | 3.3 |
| $R_{pim}$ | 0.067 (0.542) |
| I / σ (I) | 5.4 (1.5) |
| $CC_{1/2}$ | 0.995 (0.564) |
| Completeness (%) | 98.8 (99.9) |
| | |
| **Refinement** | |
| Resolution (Å) | 65.777-2.566 (2.610-2.566) |
| No. reflections | 89354 (4483) |
| $R_{work}/R_{free}$ | 22.0/26.3 |
| | |
| No. atoms | 6454 |
| Protein | 6359 |
| Ligand/ion | 95 |
| B-factors | 69.0 |
| Protein | 63.41 |
| Ligand/ion | 56.38 |
| R.m.s deviations | |
| Bond lengths (Å) | 0.003 |
| Bond angles (°) | 0.61 |
| Ramachandran plot | |
| % favored | 97.70 |
| % allowed | 2.30 |
| % outliers | 0.00 |
| Molprobity | |
| Clashscore | 2.72 |

*Values in parentheses are for the highest-resolution shell.
